# Discovering General Multidimensional Associations

**DOI:** 10.1371/journal.pone.0151551

**Published:** 2016-03-18

**Authors:** Ben Murrell, Daniel Murrell, Hugh Murrell

**Affiliations:** 1 Department of Medicine, University of California San Diego, San Diego, United States of America; 2 Centre for Molecular Informatics, Department of Chemistry, University of Cambridge, Cambridge, United Kingdom; 3 Computer Science, University of KwaZulu-Natal, Pietermaritzburg, South Africa; Memorial Sloan Kettering Cancer Center, UNITED STATES

## Abstract

When two variables are related by a known function, the coefficient of determination (denoted *R*^2^) measures the proportion of the total variance in the observations explained by that function. For linear relationships, this is equal to the square of the correlation coefficient, *ρ*. When the parametric form of the relationship is unknown, however, it is unclear how to estimate the proportion of explained variance equitably—assigning similar values to equally noisy relationships. Here we demonstrate how to directly estimate a generalised *R*^2^ when the form of the relationship is unknown, and we consider the performance of the Maximal Information Coefficient (MIC)—a recently proposed information theoretic measure of dependence. We show that our approach behaves equitably, has more power than MIC to detect association between variables, and converges faster with increasing sample size. Most importantly, our approach generalises to higher dimensions, estimating the strength of multivariate relationships (*Y* against *A*, *B*, …) as well as measuring association while controlling for covariates (*Y* against *X* controlling for *C*). An R package named *matie* (“Measuring Association and Testing Independence Efficiently”) is available (http://cran.r-project.org/web/packages/matie/).

## Introduction

Measures of association between variables are useful across the sciences. Often the form of the association is unknown and traditional methods for association mining that assume particular functional forms (usually linearity) can miss or underestimate associations that are non-linear. When large numbers of variables are examined and where associations are expected to be complex, as is common in the biological sciences in particular, tools that *automatically* quantify associations without restrictive assumptions are particularly useful, since the number of pairwise associations grows quadratically with the number of variables, and higher order relationships grow even faster. Thus, finding good methods for association mining should be prioritised.

Reshef *et al*. describe [1] desired properties of a measure of bivariate association: generality and equitability. A measure that is general will discover, with sufficient sample size, any departure from independence, while a measure that is equitable will assign similar scores to equally noisy relationships of different kinds. A further attractive property is that a measure should scale like the coefficient of determination (*R*^2^): the proportion of variance explained.

Reshef *et al*. demonstrate [1] that other measures of association (including Spearman’s rank correlation, mutual information, maximal correlation and principal curve-based correlation) are not equitable; different functional forms with similar amounts of noise can produce vastly different estimates of association strength.

Here, we explore a conceptually simple approach to quantifying and testing for relationships between variables, showing that generality and equability can be achieved by estimating a generalised *R*^2^ through density approximation. We first describe the approach, then proceed to benchmark it on multiple simulated scenarios, examining how it behaves and what power it has as a statistical test. We demonstrate some desirable properties of our method, including the ability to characterise higher order associations between more than two variables, and we highlight some pathologies of MIC.

First consider a function with additive noise, y=f(x)+N. The coefficient of determination is the proportion of variance in *y* “explained” by the deterministic component *f*(*x*) relative to the total variance in *y*, which is inflated by unexplained stochastic noise, N. This notion of variance is defined in terms of average squared deviations—the distance between a data point and a model. The explained variance, *R*^2^, is $1-\sigma_{Error}^{2}/\sigma_{Total}^{2}$, where the total variance ($\sigma_{Total}^{2}$) is the average squared deviation from a flat “null” model and the error variance ($\sigma_{Error}^{2}$) is the average squared deviation from *f*(*x*), the “alternative” model.

Least squares regression assumes that observations are normally distributed about the explanatory function. The deviation of a point from the regression line can thus be expressed as a probability density, and *R*^2^ has an equivalent form [2–4]:

$$\begin{array}{c} R^{2}=1-\prod_{i}{\left(\frac{P(x_{i},y_{i}|null)}{P(x_{i},y_{i}|alt)}\right)}^{2/n} \end{array}$$ (1)

**Fig 1:**
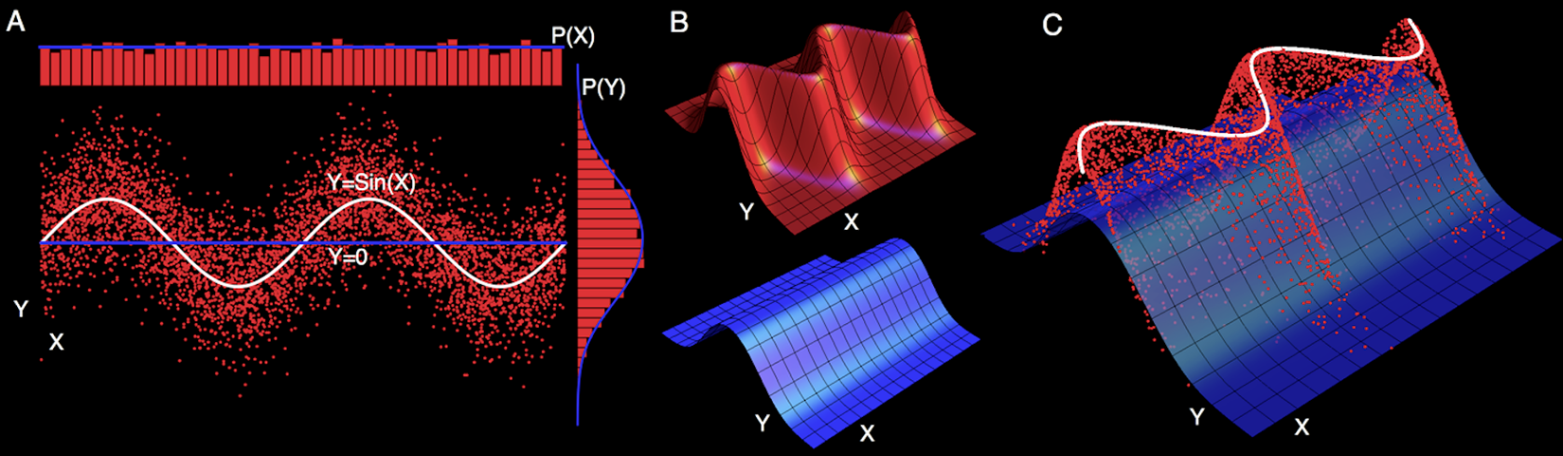
Illustrating the generalised **R**^**2**^. Panel **A**: Data is normally distributed about the alternative model—the white regression line *Y* = sin(*X*). The null model is the blue *Y* = 0. Marginal distributions of *X* and *Y* are represented above and to the right. The classical *R*^2^ is calculated using deviations of the samples from the blue and white lines. Panel **B** depicts the probability distribution over *x*_*i*_, *y*_*i*_ for the alternative (red) and null (blue) models. Panel **C** shows the height of the observations on the alternative distribution, relative to the null distribution. The generalised *R*^2^ is calculated from the ratio of these heights, and does not require an explicit regression line (white), which is included only as a guide for the eye. See section 2 of S1 File for numerical examples of varying noise levels.

This formulation of *R*^2^ asserts that the proportion of *unexplained* variance is the geometric mean of the squared ratio of the probability of observing a data point under the null model over the probability of that data point under the alternative model. The explained variance is 1 minus the unexplained proportion. See Fig 1 for a visual depiction and see section 1 of S1 File for a derivation.

Since this *R*^2^ now depends only on the probability density ratio between two models, it is applicable even when the assumptions behind least squares regression are violated. This is a powerful rethinking of *R*^2^. The idea of “explained variance” is generalised away from the restrictive assumptions of normally distributed noise, and, most importantly, the very notion of a regression curve is no longer required. This generalised *R*^2^ can be calculated as long as the probability distributions for the null and alternative models can be evaluated.

We base our measure of dependence between variables upon this generalised *R*^2^. Even when a known distribution generates our data, we still need to specify the null distribution before *R*^2^ can be computed, but this generalised definition of *R*^2^ is agnostic about a choice of null model. An attractive property for a measure of dependence is that it is 0 if and only if *X* and *Y* are independent. A sensible choice of null model is thus where *P*(*X*, *Y*) = *P*(*X*)*P*(*Y*), enforcing independence. Since explicitly choosing a null distribution places a restriction on the generalised *R*^2^, we distinguish our measure of association, calling it *A*. Classical *R*^2^ from least squares regression assumes a different choice of null model (a constant function with normally distributed errors), so *A* can be thought of as a sister to classical *R*^2^. They are equivalent for bivariate Gaussian distributions where the marginals are also normally distributed, but will differ when the null model for classical *R*^2^—a constant function with Gaussian errors—is a particularly bad fit (see section 3 of S1 File). *A* also has an information theoretic interpretation: for known distributions it is a sample estimate that converges to Linfoot’s ‘Informational Measure of Correlation’ [5] when the number of observations tends to infinity (see section 4 of S1 File).

**Fig 2:**
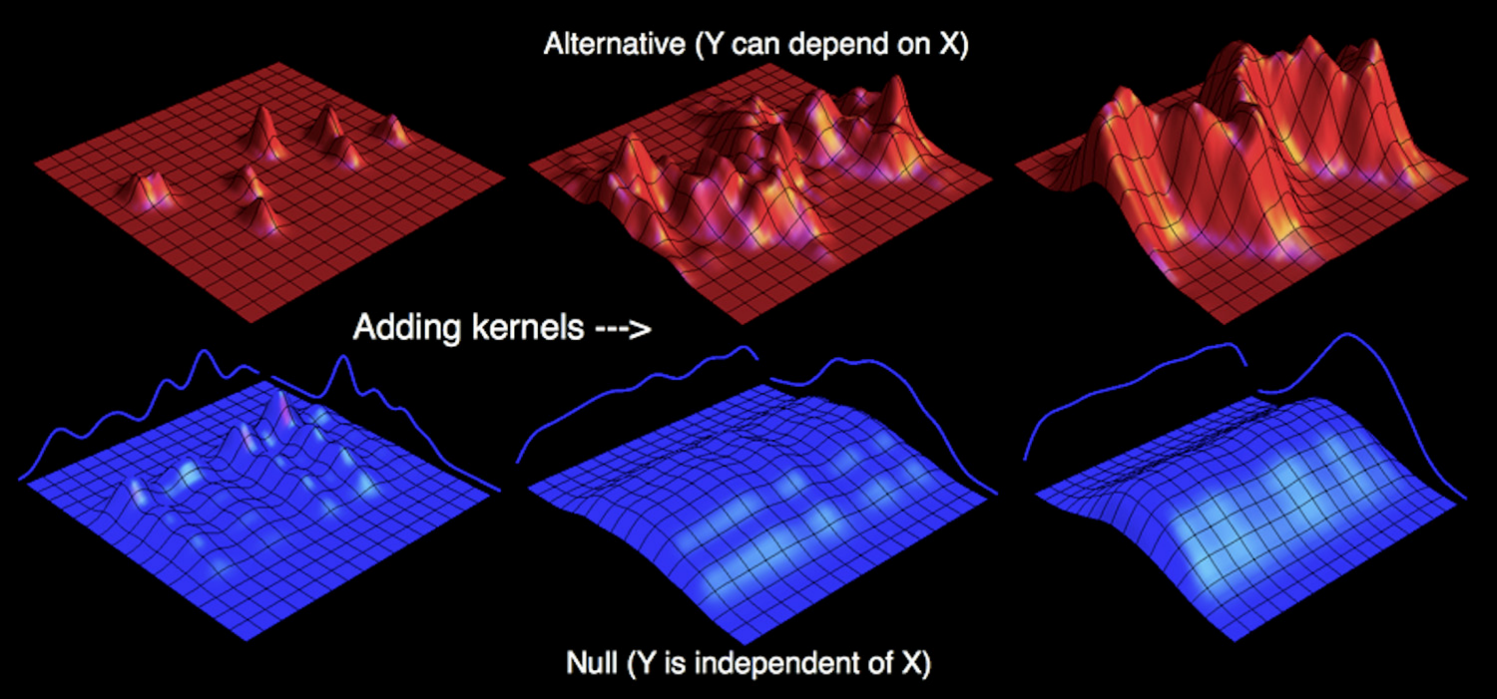
Estimating an unknown distribution. The distribution for the alternative model (red—where *X* can depend on *Y*) is constructed by adding two dimensional Gaussian “kernel” distributions centered at each observation. As more of these kernels are added, the distribution comes to resemble the true distribution from which the observations are sampled. We can use a similar approach to estimating a null model that expressly disallows any dependence between *X* and *Y* (blue) by constructing one dimensional marginal distributions (the blue lines to either side) by summing one dimensional Gaussian kernels, and then creating the joint distribution as the product of these estimated marginals.

So far, the computation of *A* requires a known distribution. Estimating $\hat{A}\approx A$ for a number of observations from an unknown distribution thus reduces to the problem of estimating the density at each point for an independent null and (potentially) dependent alternative model. We adopt a kernel density approach [6, 7], where the density of the distribution at each point is approximated by the sum over a number of Gaussian ‘kernel’ distributions centered at nearby points (see Fig 2). For the null model, we constrain the joint density to be the product of estimates of the marginal densities, enforcing independence. We wish to constrain $\hat{A}$ to vary between 0 and 1, so we cannot allow the null to outperform the alternative model, lest $\hat{A}$ become negative. We thus define the density of the alternative model at each sample point to be a weighted mixture of dependent (full joint) and independent (product of marginal) models, with a single mixture parameter controlling the proportion for all points. Therefore, the alternative model can reduce to the null model as a special case, ensuring non-negativity. We estimate the model parameters—and thus the densities—by maximizing the cross-validation likelihood (see Methods).

We also introduce a statistical test for non-independence associated with $\hat{A}$, computing *p*-values through a randomization procedure (See S1 File section 6 for details). Briefly, we use the cross-validation likelihoods for both the null and alternative models to produce a cross-validation likelihood ratio statistic (cvLRS). The null test statistic distribution can be obtained by randomly permuting the variables to break the association and induce independence, but we provide a fast approximation to this distribution for bivariate relationships, obtained by fitting a mixture of *χ*^2^ distributions to the empirical permutation distribution.

As pointed out in Speed [8], an important question is how much of the variance in *Y* can be explained by *X*, after controlling for *C*. Here, we introduce a non-linear analogue of the semipartial correlation coefficient, which is one approach to ‘controlling’ for covariates in the linear model setting. In the linear case, the semipartial (squared) correlation between Y and X controlling for C, denoted here as $R_{Y,X;C}^{2}$, is the proportion of variance in Y that can be uniquely explained by X, after the contribution of C has been accounted for. This has a simple interpretation in terms of the coefficients of determination: $R_{Y,X;C}^{2}=R_{Y,XC}^{2}-R_{Y,C}^{2}$, where $R_{Y,XC}^{2}$ is the proportion of variance in *Y* accounted for by *X* and *C* together, and $R_{Y,C}^{2}$ is the proportion of variance in *Y* accounted for by *C* alone. Here, we consider the analogous quantity defined in terms of our nonlinear measure of association: ${\hat{A}}_{Y,X;C}={\hat{A}}_{Y,XC}-{\hat{A}}_{Y,C}$.

## Results

**Fig 3:**
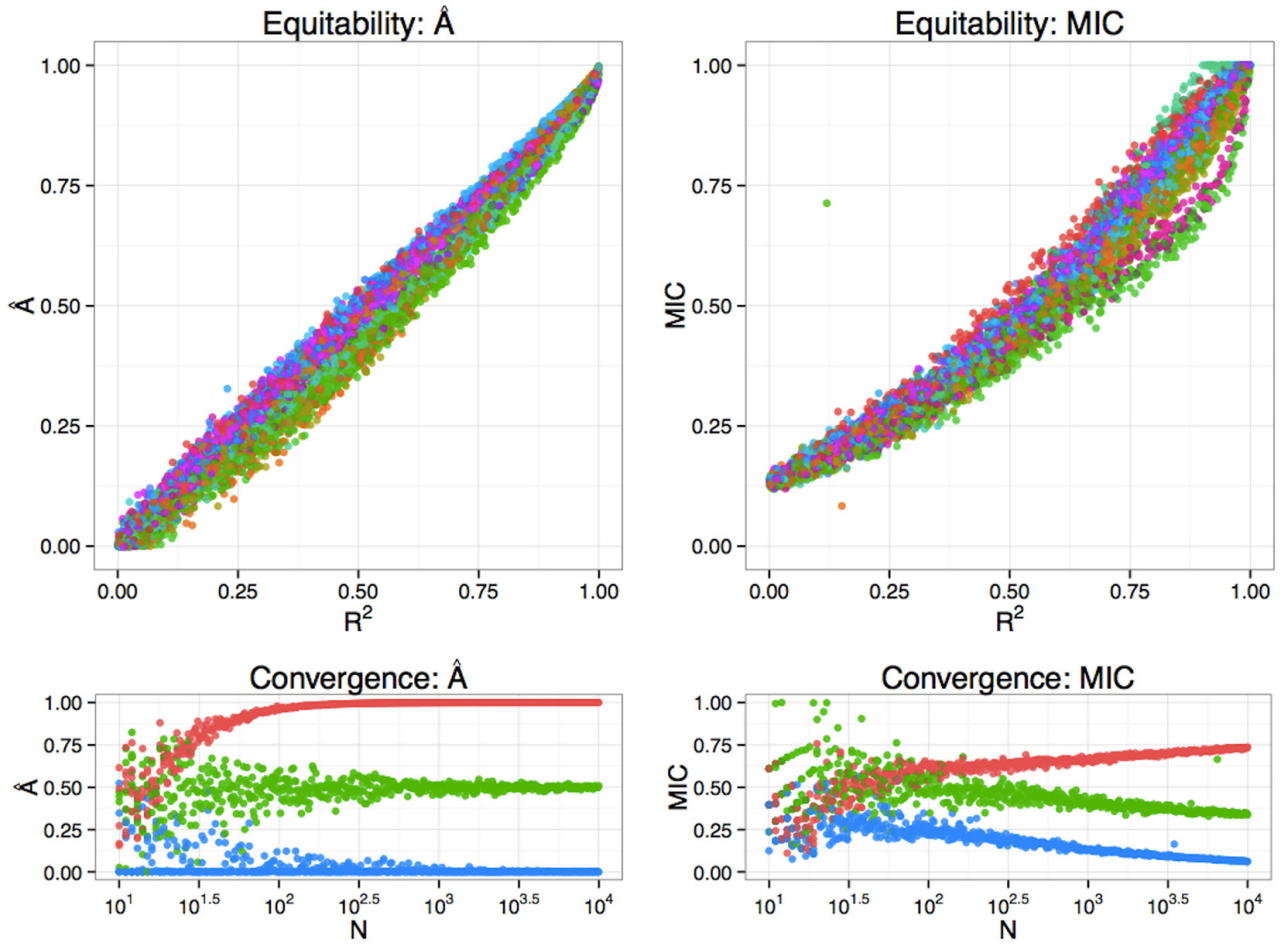
Equitability and convergence of $\hat{\text{A}}$. Top: For functions of 2 variables, $\hat{A}$ is approximately equitable, as demonstrated with 16 example functions, with *N* = 1000. Each function is marked with a different color. $\hat{A}$ (left) is closer to the classical *R*^2^ than MIC (right), especially for associations near independence. Bottom: Estimates of association from $\hat{A}$ (left) and MIC (right) as sample size (*N*) increases for three different relationships: a noiseless circle (red), a bivariate normal distribution with expected *R*^2^ = 0.5 (green), and independent noise (blue). MIC converges very slowly.

Fig 3 demonstrates that $\hat{A}$ is approximately equitable across a number of relationships (see S1 File section 5 for details of the relationships tested—the sample size was *n* = 1000 in all cases), and is in greater agreement with classical *R*^2^ than is MIC, especially for relationships where *R*^2^ is close to 0. The noise model was Gaussian in all simulations, and other noise models could exhibit different behavior. When the marginal distribution of a variable departs substantially from a normal distribution, $\hat{A}$ (like MIC) may produce more conservative estimates of association than classical *R*^2^ (see S1 File section 3). This is because the null model for $\hat{A}$ makes less restrictive assumptions (only independence is assumed, without a parametric form), describing the data better than the null model for classical *R*^2^, which is a constant function with Gaussian errors.

$\hat{A}$ converges faster with increasing sample size than MIC (Fig 3—bottom panels). For example, despite having a theoretical large-sample limit of 1 for a noiseless circle [1], *MIC* ≈ 0.74 when *N* = 10000. In contrast, $\hat{A}≳0.99$ when *N* ≥ 200.

When testing for significance of non-linear associations, we should expect no free lunch—the power of each method could vary depending on the form of the relationship. Methods should be chosen based on their performance on the kinds of relationships expected from the empirical data. Doing this rationally would require a thorough characterisation of the space of possible relationships which is beyond the scope of this paper. We do, however, compare four tests on 7 different relationships, for illustrative purposes (See S1 File section 6 for details), and find the $\hat{A}$ significance test to be attractive. It has greater power to detect associations than MIC for all but one of the relationships we tested, and outperforms Székely’s dCov test for association [9] for all non-linear relationships tested. The $\hat{A}$ test was comparable in performance to the recently proposed HHG test [10], having greater power on 4 out of 7 tested relationships, being particularly more powerful for circular relationships, and low-noise high-complexity non-linear relationships potentially embedded in background noise. We note that MIC has a user-tunable parameter, and this has recently been shown to influence its power to detect relationships [11]. Our results were obtained with the default MIC parameter settings, and may vary with different parameter choices.

**Fig 4:**
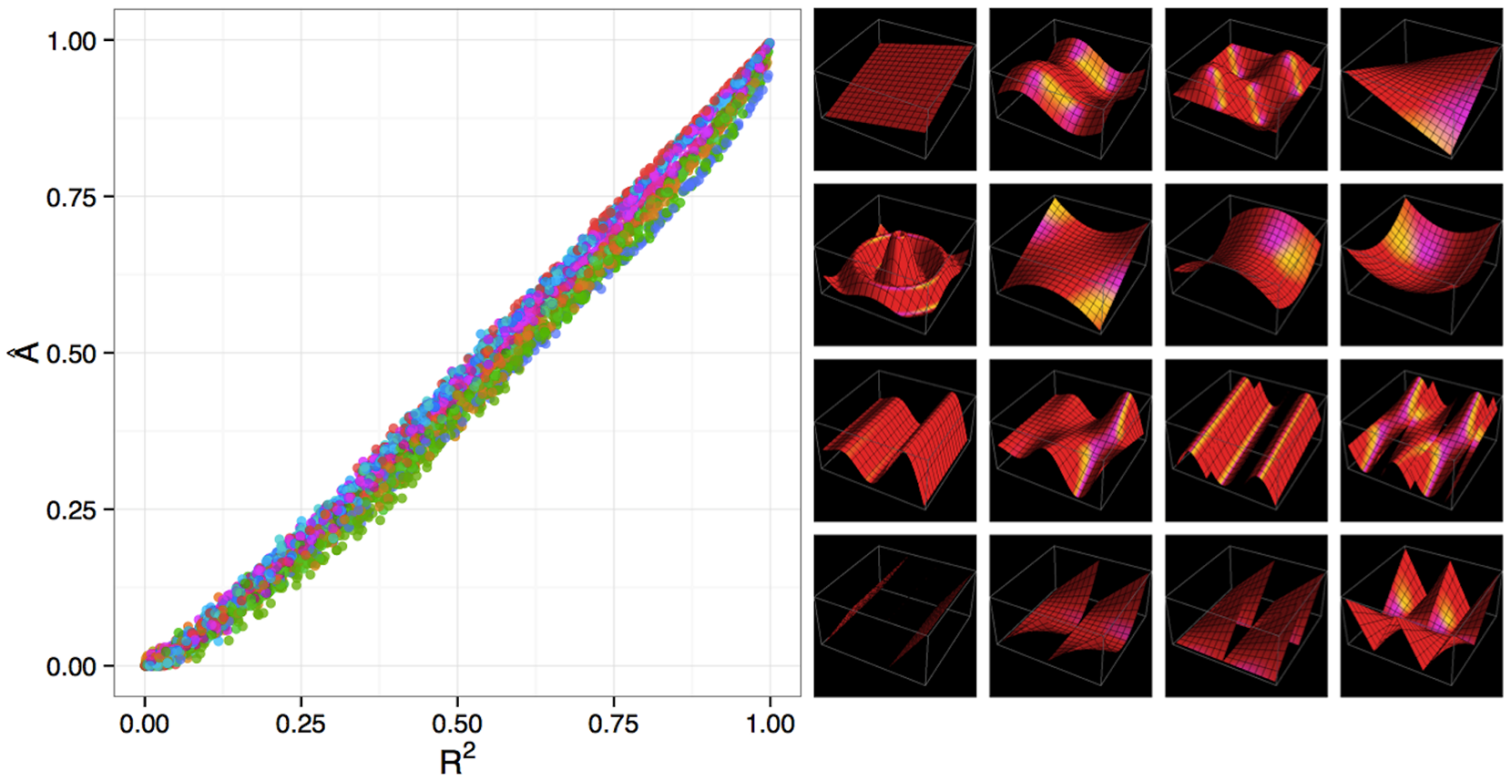
Equitability of $\hat{\text{A}}$ in higher dimensions. $\hat{A}$ against *R*^2^ (left) for multivariate datasets (*N* = 4000 for all) generated by adding normally distributed noise to 16 different functions of two variables (right—see S1 File section 5 for more detail).

As shown in Fig 4, $\hat{A}$ generalises well to multiple dimensions, producing equitable estimates very similar to classical *R*^2^ for functions of two dimensions. If desired, it can assess the strength of association between vector valued variables, indicating what proportion of the variance in (*X*, *Y*) is explained by (*A*, *B*, *C*), for example. It also generalises to more than two variables (with each variable being possibly vector valued), which could be used to discover lower dimensional manifolds embedded in a higher dimensional space (see S1 File section 7). We do observe that performance degrades in higher dimensions (data not shown), with $\hat{A}$ underestimating the association we would expect from a fixed *R*^2^ as the dimensionality increases, which is likely a result of the inefficiency of kernel density estimation in higher dimensions.

We show (see S1 File section 8) that our ability to control for covariates is well behaved, agreeing with the linear semipartial correlation when all relationships are linear. Semipartial association becomes more interesting when the data are non-linear. When relationships are non-linear, the standard linear semipartial correlation can severely underestimate semipartial association (only seeing the linear dependence of *Y* upon *X*, regardless of the control variable *C*) but, more interestingly, it can also overestimate the semipartial association between *Y* and *X* by ignoring a non-linear dependence of *Y* on the control variable *C*, returning values close to 1 when in fact *Y* is conditionally independent of *X* given *C*. Our non-linear semipartial association has no such difficulty, returning values close to 0 for such cases.

**Fig 5:**
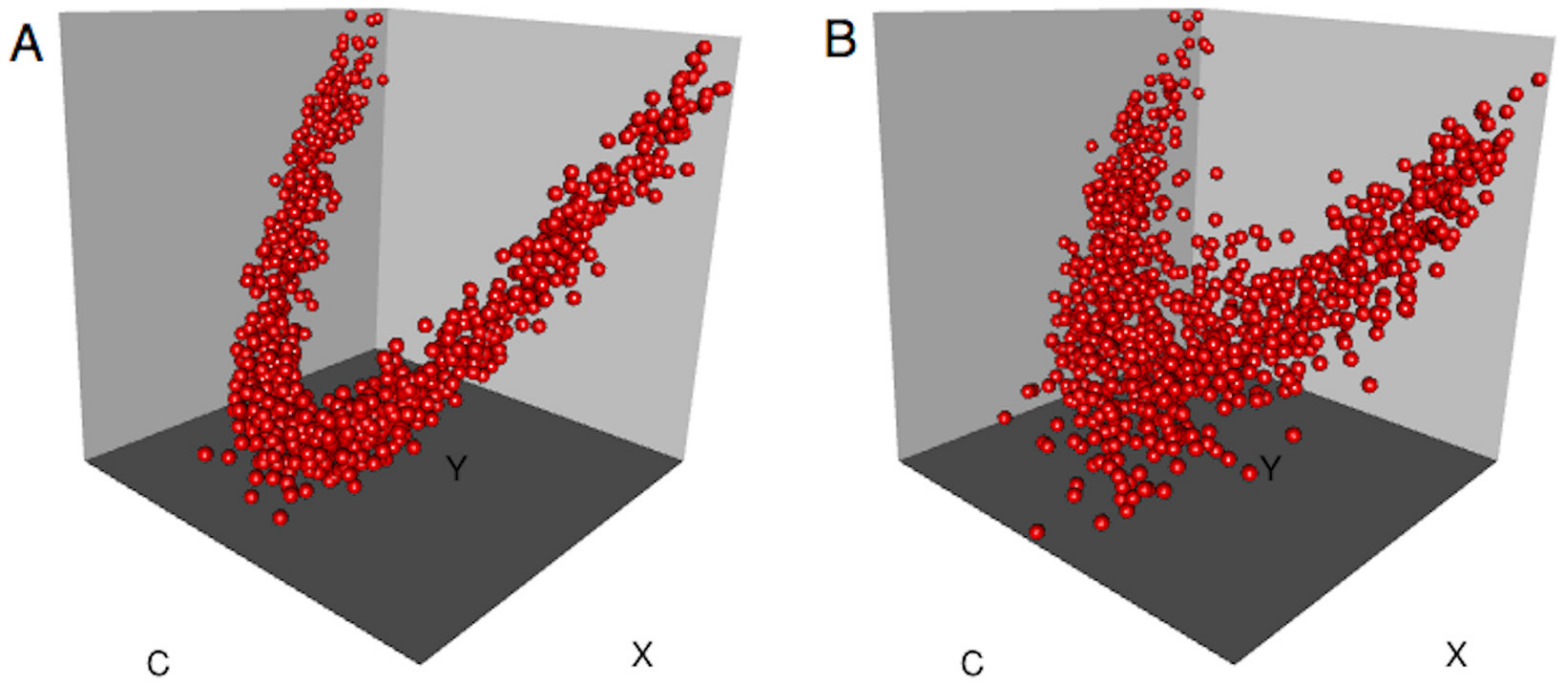
Semipartial correlation examples. In panel **A**, *Y* is conditionally independent of *X* given *C*. Missing the quadratic dependence of *Y* on *C*, linear $R_{Y,X;C}^{2}=0.90$. Non-linear semipartial association correctly identifies *C* as explaining all of the variance in *Y*, yielding ${\hat{A}}_{Y,X;C}=0$. In panel **B**, *Y* also depends on the noise term in *X* (see text), and non-linear semipartial association captures this: ${\hat{A}}_{Y,X;C}=0.28$.

Consider, for example, the following data generating process (Fig 5, panel **A**):

$$\begin{array}{ccc} C & ∼ & U(-1,1)\\ X & ∼ & C^{2}+N\left(0,\epsilon \right)\\ Y & ∼ & C^{2}+N\left(0,\epsilon \right) \end{array}$$

*Y* depends quadratically on the control variable *C*, and is conditionally independent of *X* given *C*, so discovering *X* tells us nothing about *Y* if we already know the value of *C*. *X* and *Y* are strongly linearly related, but all the association can be explained through the common influence of *C*. When computing the linear semipartial correlation $R_{Y,X;C}^{2}$, *Y* appears to depend strongly on *X* even after controlling for *C* ($R_{Y,X;C}^{2}=0.90$), but this is because the quadratic dependence of *Y* upon *C* is missed by a linear model. The non-linear semipartial association discovers the quadratic dependence of *Y* on *C*, and correctly declares that *X* provides no additional influence: ${\hat{A}}_{Y,X;C}=0$. If we “correct” the *linear* semipartial correlation by controlling for *C*^2^ rather than *C*, transforming the association back into linearity, $R_{Y,X;C^{2}}^{2}=3.5\times 10^{-5}$, validating the nonlinear semipartial association of 0.

By contrast, consider the following process (Fig 5, panel **B**):

$$\begin{array}{ccc} C & ∼ & U(-1,1)\\ X & ∼ & C^{2}+N\left(0,\epsilon_{2}\right)\\ Y & ∼ & X+N(0,\epsilon ) \end{array}$$

Again, there is a quadratic dependence between *Y* and *C*, but this time *Y* also depends partly on the noise component in *X*, which means that, while *C* can account for much of the variance in *Y*, *C* cannot account for all of it. The linear semipartial correlation again misses the quadratic dependence of *Y* on *C*, incorrectly deducing that *C* cannot account for any of the variance in *Y*, and asserting the *X* explains almost all of the variance in *X* ($R_{Y,X;C}^{2}=0.90$). The non-linear semipartial association behaves sensibly, asserting that 28% of the variance in *Y* is explained by *X* after controlling for *C* (${\hat{A}}_{Y,X;C}=0.28$). This result is appropriate given the value of the linear semipartial correlation correcting for the quadratic dependence on *C* (using *C*^2^ as the control variable): $R_{Y,X;C^{2}}^{2}=0.28$.

Section 7 of S1 File demonstrates the use of $\hat{A}$ on a dataset of relationships obtained from Markov-Chain Monte Carlo samples from a phylogenetic analysis of Influenza evolution [12].

## Discussion

While this paper represents the initial practical contribution, further work remains to characterise the theoretical properties of *A* and $\hat{A}$. *A* is clearly invariant to monotonic transformations of variables, but its estimate $\hat{A}$ is not, although it may be as *N* tends to infinity (in our particular implementation, we artificially enforce it through a rank transformation). Simulations suggest that $\hat{A}$ tends to 0 wherever variables are independent, and 1 whenever a relation is noiseless and nowhere flat, but perhaps there are other circumstances under which 1 will be the large sample limit (MIC, for example, can achieve 1 at large samples for noisy relationships—see S1 File section 10). Is the $\hat{A}$ test for independence consistent against all alternatives, achieving a power of 100% as *N* tends to infinity whenever independence is violated in any way? $\hat{A}$ appears to be robust to outliers (see S1 File section 11), but is it possible to design outlier distributions that mislead it? $\hat{A}$ could also be improved by more sophisticated techniques to estimate the density ratio of the joint and independent distributions [13, 14], which may improve the convergence of $\hat{A}$ for smaller sample sizes, but at a computational cost. *A* can be viewed as a transformed mutual information, and the performance and equitability of mutual information estimators needs to be examined and compared to our approach for estimating $\hat{A}$, but this is beyond the scope of the present paper. Also, it may be possible to use our approach for estimating $\hat{A}$ as a means of estimating mutual information itself. This could turn out to perform similarly to kernel density-based estimates of mutual information (especially [15] which also uses cross-validation to estimate the kernel bandwidth), but none of those, to our knowledge, use the mixture approach we employ here when estimating the alternative model, so some differences are expected.

Kinney and Atwal [16] have recently asserted that “No nontrivial dependence measure can satisfy *R*^2^-equitability”, providing a theorem to support this. However, as we show [17], their result hinges on a peculiar definition of “noise”, which allows a trend to be embedded in the noise term, effectively introducing an un-identifiability in their definition which they then exploit to prove the notion incoherent. When you take the trend out of the noise term, you are left with a perfectly sensible notion of “*R*^2^-equitability”. Under the resulting notion of “*R*^2^-equitability”, however, *R*^2^ is sensitive to non-linear transformations of variables, and isn’t symmetric, as one would expect of any measure defined in terms of squared departure from a trend line. Here we have shown how to inherit the good properties of measures of dependence that are based on the ratio of full joint over independent distributions, which are not sensitive to transformations of variables and are symmetric, while still behaving like an *R*^2^, and giving results similar (but not identical) to canonical *R*^2^ for reasonable bivariate relationships.

## Methods

Consider two (possibly) vector valued variables, **X** and **Y**, with *n* observations {**x**_1_, …, **x**_*n*_} and {**y**_1_, …, **y**_*n*_}. Each **x**_*i*_ itself may be a vector $x_{i}^{a},\ldots ,x_{i}^{z}$, as may each **y**_*i*_. Further, imagine three kernel distributions, *K*_*X*_(**x**), *K*_*Y*_(**y**) and *K*_*XY*_(<**x**,**y** >), where the kernels are symmetric, non-negative, and integrate to 1, and where angle brackets indicate vector concatenation. Our null model assumes that **X** and **Y** are independent, and so we define the leave-one-out cross validation likelihood as the product of marginal kernel density estimates:

$$\begin{array}{c} L_{CV}\left(null\right)=\prod_{i=1}^{n}P\left({\text{x}}_{i}|{\text{x}}_{\forall j\neq i}\right)P\left({\text{y}}_{i}|{\text{y}}_{\forall j\neq i}\right) \end{array}$$ (2)

$$\begin{array}{c} \approx \prod_{i=1}^{n}[\sum_{\forall j\neq i}\frac{K_{X}\left({\text{x}}_{j}-{\text{x}}_{i}\right)}{n-1}\times \sum_{\forall j\neq i}\frac{K_{Y}\left({\text{y}}_{j}-{\text{y}}_{i}\right)}{n-1}] \end{array}$$ (3)

The alternative model allows **Y** to depend on **X** for a proportion of points, (1 − *w*), with a leave-one-out cross validation likelihood defined as:

$$\begin{array}{c} L_{CV}\left(alt\right)=\prod_{i=1}^{n}[\left(1-w\right)\times P\left({\text{x}}_{i},{\text{y}}_{i}|{\text{x}}_{\forall j\neq i},{\text{y}}_{\forall j\neq i}\right)+w\times P\left({\text{x}}_{i}|{\text{x}}_{\forall j\neq i}\right)P\left({\text{y}}_{i}|{\text{y}}_{\forall j\neq i}\right)] \end{array}$$ (4)

$$\begin{array}{c} \approx \prod_{i=1}^{n}[\left(1-w\right)\sum_{\forall j\neq i}\frac{K_{XY}\left(<{\text{x}}_{j}-{\text{x}}_{i},{\text{y}}_{j}-{\text{y}}_{i}>\right)}{n-1}+w\;\sum_{\forall j\neq i}\frac{K_{X}\left({\text{x}}_{j}-{\text{x}}_{i}\right)}{n-1}\sum_{\forall j\neq i}\frac{K_{Y}\left({\text{y}}_{j}-{\text{y}}_{i}\right)}{n-1}] \end{array}$$ (5)

In our particular implementation, the values of each variable are replaced with their ranks (this is for computational convenience and should have little effect since *A* itself is invariant to order preserving transformations of variables) with ties broken randomly, and the kernels are isotropic Gaussians, with *K*_*X*_ and *K*_*Y*_ sharing an ‘independent’ kernel variance parameter $\sigma_{I}^{2}$, and *K*_*XY*_ having a distinct ‘dependent’ variance parameter, $\sigma_{D}^{2}$. With the rank-transformed observations, the data are now integers, and we discretize our Gaussian kernels by evaluating them on a bounded grid, and re-normalizing them. The null model thus has a single parameter, $\sigma_{I}^{2}$, and the alternative model has 3 parameters: $\sigma_{I}^{2}$, $\sigma_{D}^{2}$, and *w*. These parameters are optimised numerically to maximise the cross-validation likelihood, yielding $\hat{A}$ after employing Eq (1). We found this estimate to be slightly biased down (when calibrated relative to classical *R*^2^ for bivariate Gaussians, where *A* = classical *R*^2^) for small samples, so we included an empirically-estimated small samples correction (see S1 File section 12). An R package named *matie* (“Measuring Association and Testing Independence Efficiently”—see S1 File section 13) for estimating $\hat{A}$ is available on CRAN (http://cran.r-project.org/web/packages/matie/). For efficiency, core routines, such as the leave-one-out cross-validation likelihood calculation, are written in C, with everything else in R. The optimization itself is done numerically, using the *dfoptim* package. Like MIC, estimating $\hat{A}$ is quadratic in the sample size, but with a much lower growth rate than MIC (see S1 File section 14).

## Supporting Information

S1 FileSupporting text, figures and tables.
Generalized *R*^2^ generalizes classical *R*^2^.Sinusoidal example.Classical *R*^2^, generalized *R*^2^, A, $\hat{A}$ and Linfoots Informational Measure of Correlation.A is a sample approximation of Linfoots Informational Measure of Correlation.List of functions used for the equitablity plots.Significance tests.$\hat{A}$ can detect manifolds.Semipartial association—controlling for variables.Example: BEAST analysis.MIC can return 1 for noisy relationships.$\hat{A}$ is robust to outliers.A small samples bias correction.matie, An R package for computing $\hat{A}$Execution time: matie versus MIC.
(PDF)
