# Supplementary material for: Discovering General Multidimensional Associations
Source: PLoS One. 2016 Mar 18;11(3):e0151551. doi: 10.1371/journal.pone.0151551 (PMC4798755; doi:10.1371/journal.pone.0151551)
Supplement: S1 File — Generalized R2 generalizes classical R2.Sinusoidal example.Classical R2, generalized R2, A, A^ and Linfoots Informational Measure of Correlation.A is a sample approximation of Linfoots Informational Measure of Correlation.List of functions used for the equitablity plots.Significance tests.A^ can detect manifolds.Semipartial association—controlling for variables.Example: BEAST analysis.MIC can return 1 for noisy relationships.A^ is robust to outliers.A small samples bias correction.matie, An R package for computing A^Execution time: matie versus MIC. (PDF) [file pone.0151551.s001.pdf]

Supporting Information:
Discovering General Multidimensional Associations

Ben Murrell, Daniel Murrell, Hugh Murrell

Contents

1 Generalized R^2 generalizes classical R^2 **2**

2 Sinusoidal example. **3**

3 Classical R^2, generalized R^2, A, $\hat{A}$ and Linfoot's Informational Measure of Correlation. **4**

4 A is a sample approximation of Linfoot's Informational Measure of Correlation. **9**

5 List of functions used for the equitablity plots. **10**

6 Significance tests. **12**

7 $\hat{A}$ can detect manifolds. **16**

8 Semipartial association - controlling for variables. **18**

9 Example: BEAST analysis. **19**

10 MIC can return 1 for noisy relationships. **22**

11 $\hat{A}$ is robust to outliers. **23**

12 A small samples bias correction. **25**

13 `matie`, An R package for computing $\hat{A}$. **26**

14 Execution time: matie versus MIC. **27**

1 Generalized R^2 generalizes classical R^2

Starting with Nagelkerke's generalized R^2:

$$R_N^2 = 1 - \prod_i \left(\frac{P(x_i, y_i|null)}{P(x_i, y_i|alt)} \right)^{\frac{2}{n}}$$

Given a fixed set of x_i values, and assuming that the alternate model is a regression function $y_i = f(x_i)$ with Gaussian errors and with a maximum likelihood variance estimate (or the unbiased variance estimate, with trivial changes), we have:

$$P(x_i, y_i|alt) = \frac{1}{\sigma_{alt}\sqrt{2\pi}} exp(-\frac{1}{2}\left(\frac{y_i - f(x_i)}{\sigma_{alt}}\right)^2)$$

where

$$\sigma_{alt}^2 = \frac{1}{N}\sum_i (y_i - f(x_i))^2$$

Similarily, assuming that the null model is the constant mean line, $y_i = \bar{y}$ with Gaussian errors, and with a maximum likelihood variance estimate, we have:

$$P(x_i, y_i|null) = \frac{1}{\sigma_{null}\sqrt{2\pi}} exp(-\frac{1}{2}\left(\frac{y_i - \bar{y}}{\sigma_{null}}\right)^2)$$

where

$$\sigma_{null}^2 = \frac{1}{N}\sum_i (y_i - \bar{y})^2$$

substituting in Nagelkerke's expression

$$\frac{1 - R_N^2}{\left(\frac{\sigma_{alt}}{\sigma_{null}}\right)^2} = \left(\prod_i \exp\left(\frac{1}{2}\left(\frac{y_i - f(x_i)}{\sigma_{alt}}\right)^2 - \frac{1}{2}\left(\frac{y_i - \bar{y}}{\sigma_{null}}\right)^2\right)\right)^{\frac{2}{n}}$$

taking logs

$$\log\left(\frac{1 - R_N^2}{\left(\frac{\sigma_{alt}}{\sigma_{null}}\right)^2}\right) = \frac{2}{n}\left(\sum_i \frac{1}{2}\left(\frac{y_i - f(x_i)}{\sigma_{alt}}\right)^2 - \sum_i \frac{1}{2}\left(\frac{y_i - \bar{y}}{\sigma_{null}}\right)^2\right)$$

$$\log\left(\frac{1 - R_N^2}{\left(\frac{\sigma_{alt}}{\sigma_{null}}\right)^2}\right) = \frac{2}{n}\left(\frac{N}{2} - \frac{N}{2}\right) = 0$$

thus

$$R_N^2 = 1 - \left(\frac{\sigma_{alt}}{\sigma_{null}}\right)^2 = 1 - \frac{\sum(y_i - f(x_i))^2}{\sum(y_i - \bar{y})^2} = 1 - \frac{\text{Residual Sum of Squares}}{\text{Total Sum of Squares}} = \text{Classical } R^2$$

2 Sinusoidal example.

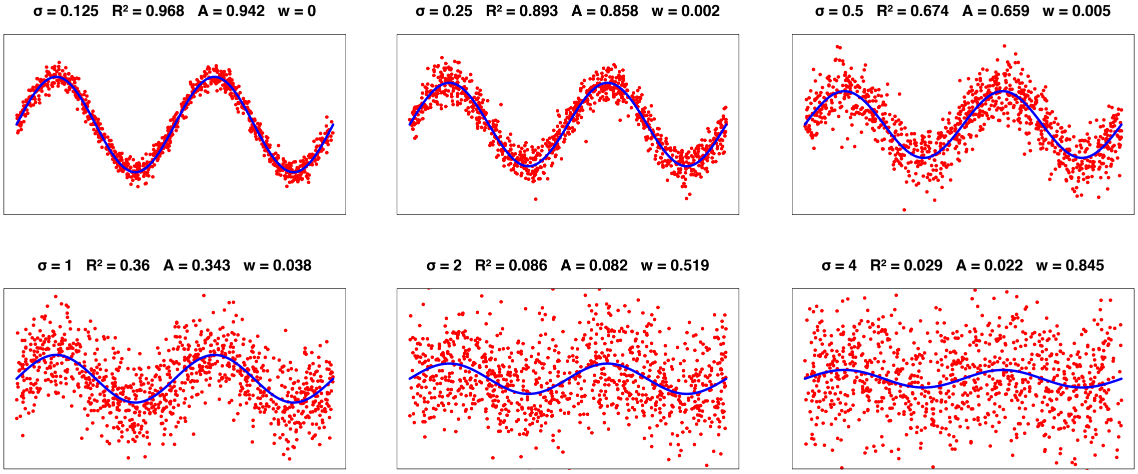

Figure 1. Sinusoidal example. To build intuition about the relationship between our measure of association and the magnitude of the variance around a function $f(x)$ (in this case, the sinusoid from fig 1 in the main text), we have generates examples with the same function ($x \sim \text{unif}(-2\pi, 2\pi)$, $y \sim \sin(x) + \mathcal{N}(0, \sigma)$), but different noise levels. Using 1000 data points, we show examples of data, titled by the standard deviation, classical R^2 (which can be calculated because $f(x)$ is known), our estimate, $\hat{A}$, and the mixture weight estimate w (see Methods of the main text for a definition of this).

3 Classical R^2, generalized R^2, A, $\hat{A}$ and Linfoot's Informational Measure of Correlation.

Figure 2 attempts to clarify the relationship between the various quantities mentioned in the main text. Firstly, there is classical R^2, which, for our purposes, is calculated as $1 - \sigma^2_{Error}/\sigma^2_{Total}$. Since the error model in classical R^2 is implicitly Gaussian (assuming least squares fitting), the regression curve, $f(x)$, is all that is required to specify the model. Generalized R^2, defined by equation 1 in the main text, relies on explicitly defined probability distributions for the null and alternative models, reducing to classical R^2 when the noise model is Gaussian with constant variance and when the null model is a flat function, $f(x) = c$. Under these conditions, calculating the generalized R^2 for the maximum likelihood parameter estimates (assuming $f(x)$ was governed by some free parameters) will yield the same result as calculating the classical R^2 with the least squares optimal parameters.

We define A to be a special case of the generalized R^2, where the null model enforces independence, and is restricted to being the product of marginal distributions. To allow the alternative model to reduce to the null as a special case, we can create the alternative to be a mixture distribution with dependent and independent components: $w \times P(X,Y|\theta) + (1-w) \times P(X|\theta)P(Y|\theta)$, where θ encodes the parameters that govern the distribution $P(X,Y|\theta)$. This reduces to the independent null model when $w = 0$ (whether or not this mixture is required – rather than just using $P(X,Y|\theta)$ alone as the alternative model – depends on your view of the requirements of a generalized R^2.). For a particular distributional form, A could be

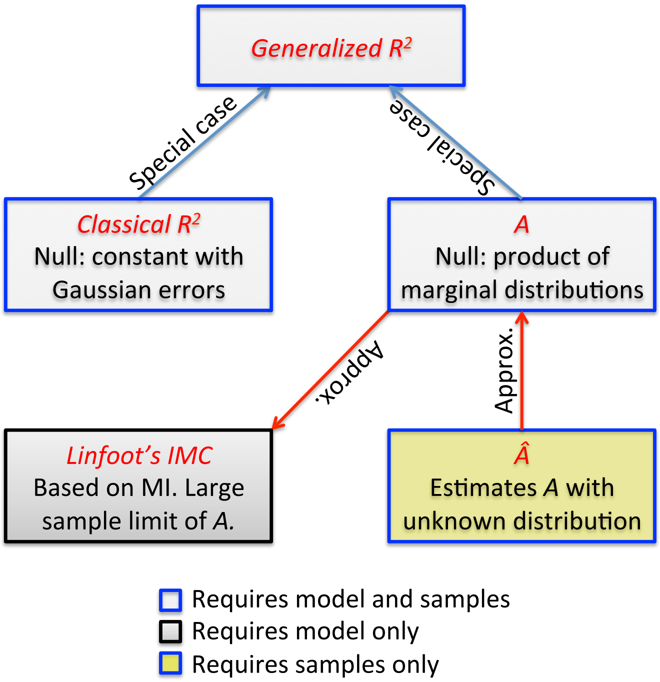

Figure 2. Relationships between measures of association. Classical R^2 and A are "sisters" – both are special cases of generalized R^2. They are equivalent for bivariate Gaussian data, but may differ for other kinds of relationships. $\hat{A}$ is the estimate of A when the distribution is not known *a priori*, and Linfoot's 'Informational Measure of Correlation', based on mutual information, is the large sample limit of A.

computed directly, although, if closed forms are not available, this may require numerical integration to compute the marginal distributions $P(X|\theta) = \int_y P(X, Y|\theta)$. The free parameters θ could be optimized by maximum likelihood, but this may be computationally intensive due to the numerical integration. (Note that estimating $\hat{A}$ using density approximation has no such difficulties.)

When the null model is selected to be a constant function with Gaussian errors, as in the context of regression, then the generalized R^2 and the classical R^2 are equivalent. Since the null for A is the product of the marginals, whenever the marginal distribution of Y departs substantially from a Gaussian (see figure 3), the null model for A will differ from the null model for classical R^2, yielding different association scores (see figure 4). Thus A and classical R^2 aren't quite estimating the same quantity, although their behavior is very similar for functions that aren't pathologically skewed (see figures 5 and 6), and they are entirely equivalent for bivariate Gaussian data.

This departure of A from classical R^2 is not undesirable. When the marginal distribution of Y is not Gaussian, then it is not sensible to use a Gaussian distribution to describe it, even under the null model (a Gaussian marginal for Y is assumed in the classical R^2). Classical R^2 is measuring how far data departs from a flat function with Gaussian errors, whereas A measures how far it departs from independence.

A depends on a parametric distribution (which may have some free parameters), and a collection of samples which are ostensibly drawn from that distribution. If the distribution is entirely fixed (ie. no free parameters), it is possible to calculate an analogous quantity directly from the distribution itself, which will be the large sample limit of A. It turns out (see SI2) that this large sample limit is equivalent to Linfoot's Information Measure of Correlation [1], which was proposed as a way to transform mutual information into an association score that was equivalent to R^2 for bivariate Gaussian distributions.

$\hat{A}$ attempts to estimate A when the distribution is unknown. The quality of this estimation will depend on the convergence behavior of the approximated densities used by $\hat{A}$ to the true generating

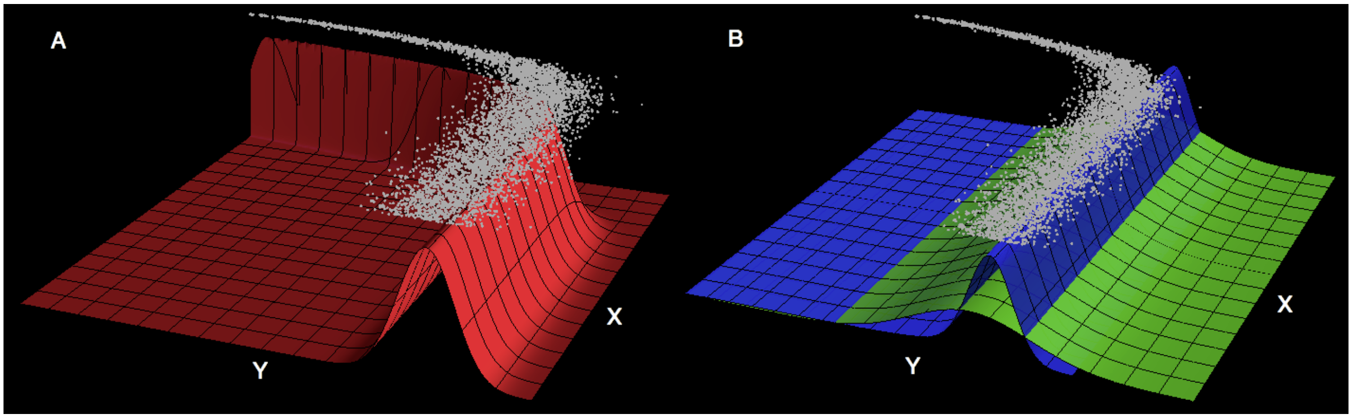

Figure 3. When A differs substantial from classical R^2 - illustration. Data (in grey) is generated from an exponential function with Gaussian noise. Panel **A** depicts the distribution that generated the data. This is the alternative model for both the classical R^2 and for A. Panel **B** depicts the null model for classical R^2 (in green), which, no matter the value of X, is a Gaussian distribution centered around the mean of the Y values. The null model for A is depicted in blue, which is the product of the marginal distributions, $P(X)P(Y)$. The null for A in blue provides a much better fit to the data than does the null model for classical R^2 (in particular, it captures the asymmetry), which causes classical R^2 to be inflated relative to A (in this example, classical $R^2 = 0.88$ and $A = 0.68$). One way of describing the difference is that classical R^2 is measuring how far data departs from a flat function with Gaussian errors, whereas A measures how far it departs from independence.

distributions. We have investigated the behavior of $\hat{A}$ by simulation, and theoretical convergence results are left for future work.

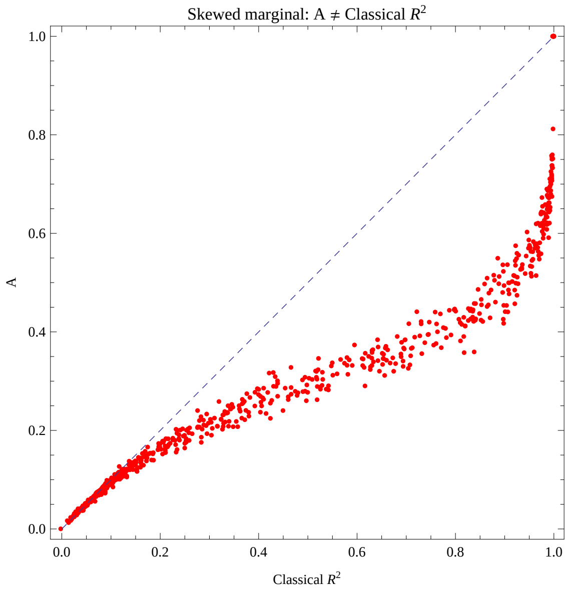

Figure 4. When A differs substantial from classical R^2 - comparison. In some cases, the marginal distribution of Y can be substantially non-normally distributed (see figure 3) and A will depart from R^2. This example is for an exponential function with additive Gaussian noise. Note that, in this section, A is calculated directly from a known distribution to illustrate this departure analytically, whereas everywhere else $\hat{A}$ is estimated from an unknown distribution.

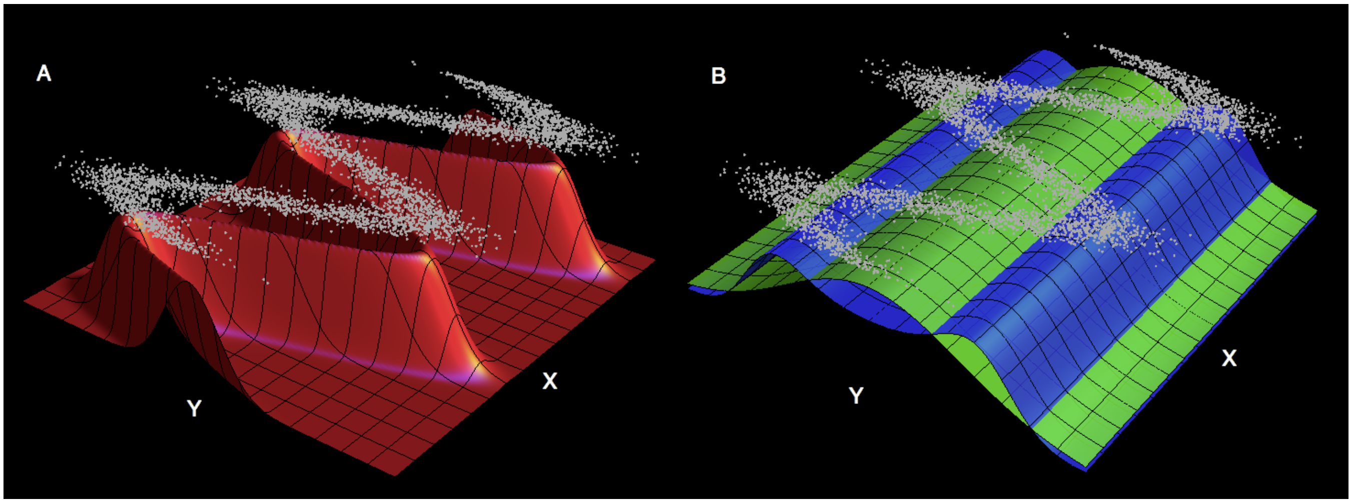

Figure 5. When A differs subtley from classical R^2 - illustration. Data (in grey) is generated from a sinusoidal function with Gaussian noise. Panel **A** depicts the distribution that generated the data. This is the alternative model for both the classical R^2, and for A. Panel **B** depicts the null model for classical R^2 (in green), which, no matter the value of X, is a Gaussian distribution centered around the mean of the Y values. The null model for A is depicted in blue, which is the product of the marginal distributions, $P(X)P(Y)$. The null for A in blue provides a better fit to the data than does the null model for classical R^2, which causes classical R^2 to be slightly inflated relative to A (in this example, classical $R^2 = 0.89$ and $A = 0.86$). The null for classical R^2 is an adequate approximation, in this case, so measuring the departure from independence, A, and the departure from a constant function with Gaussian noise, classical R^2, yields similar values.

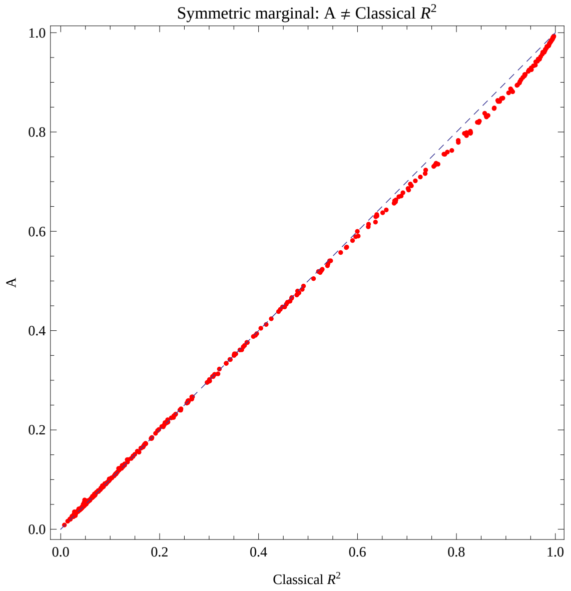

Figure 6. When A differs subtley from classical R^2 - comparison. In this case, where the data is sinusoidal, the marginal distribution of Y can be non-normally distributed, especially when there is little noise, (see figure 5) and A will depart from R^2. The degree of the departure is smaller than in the exponential case because the Gaussian marginal null model of classical R^2 is closer to the true marginal than in the exponential case. Note that, in this section, A is calculated directly from a known distribution to illustrate this departure analytically, whereas everywhere else $\hat{A}$ is estimated from an unknown distribution.

4 A is a sample approximation of Linfoot's Informational Measure of Correlation.

We assert that A (which is Nagelkerke's generalized R^2 if the null distribution is taken to be the product of the marginals of the alternative distribution), which is computed from a set of observations sampled from a known distribution M, will tend to Linfoot's IMC [1], which is based on mutual information, $I(x;y)$. When (x_i, y_i) are samples from $P(x,y|M)$, then, by the weak law of large numbers,

$$\begin{aligned} I(x;y) &= \int\int P(x,y|M) \log\left(\frac{P(x,y|M)}{P(x|M)P(y|M)}\right) dxdy \quad (1)\\ &\approx \frac{1}{n}\sum_{i=1}^{n} \log\left(\frac{P(x_i,y_i|M)}{P(x_i|M)P(y_i|M)}\right) \quad (2)\\ &\approx \frac{1}{n}\log\left(\prod_{i=1}^{n}\frac{P(x_i,y_i|M)}{P(x_i|M)P(y_i|M)}\right) \quad (3)\end{aligned}$$

Thus,

$$\begin{aligned} \text{Linfoot's IMC} = 1 - e^{-2I(x;y)} &\approx 1 - \prod_{i=1}^{n}\left(\frac{P(x_i|M)P(y_i|M)}{P(x_i,y_i|M)}\right)^{2/n} \quad (4)\\ &\approx A(x,y) \quad (5)\end{aligned}$$

5 List of functions used for the equitablity plots.

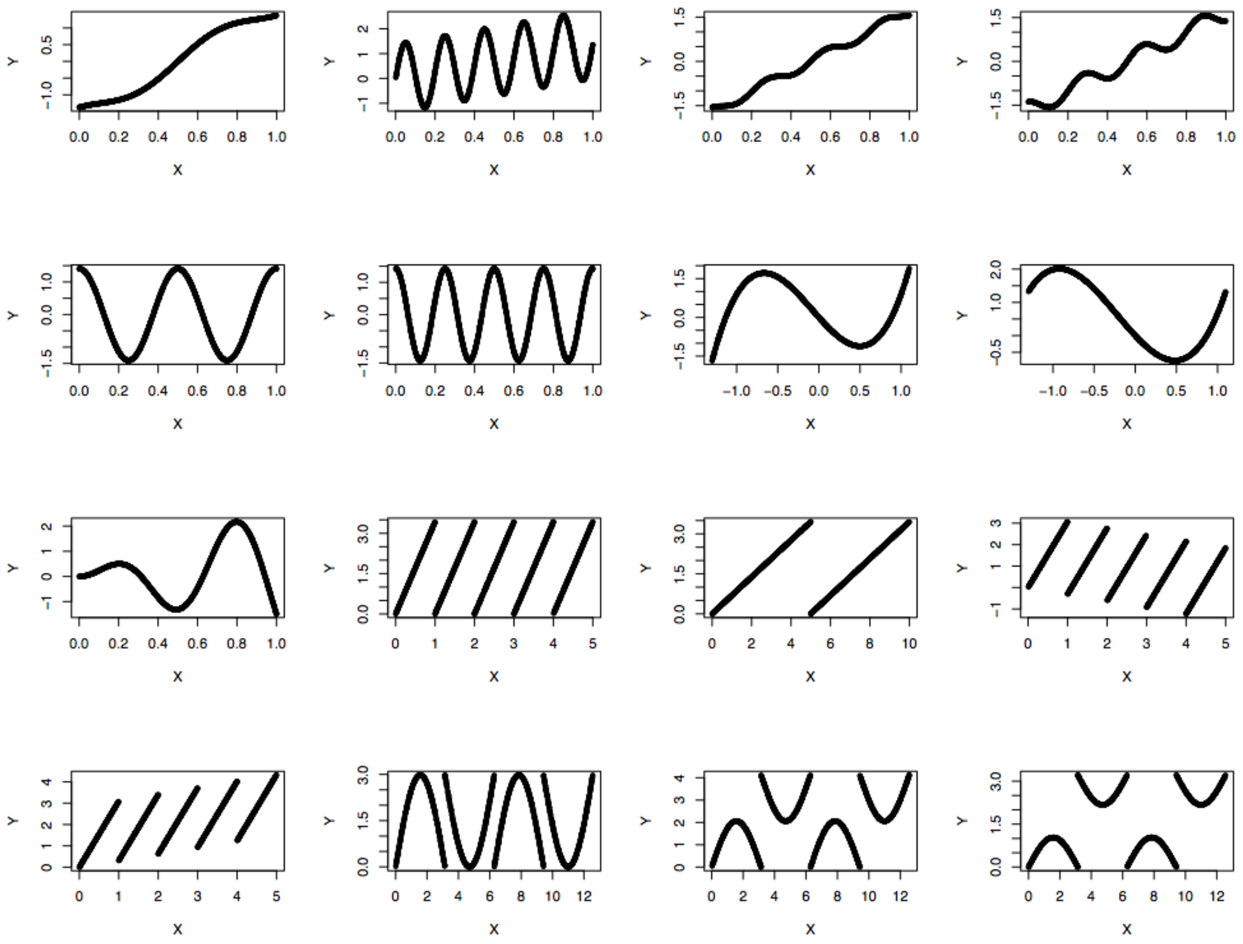

Figure 7. Functions used in equitability plots. The functions used to generate the equitability plots, before Gaussian noise is added. R code defining these functions is available from the authors upon request.

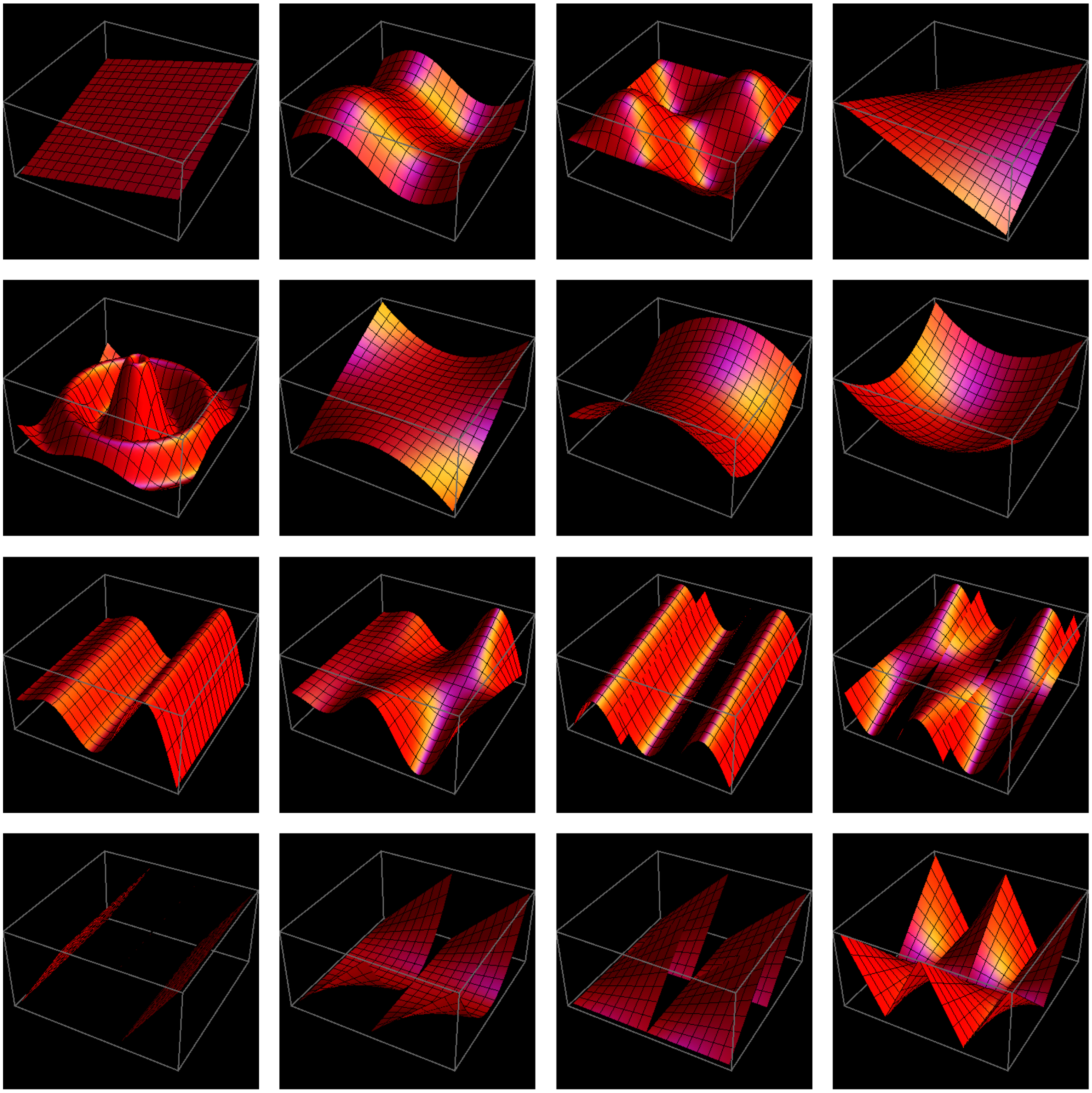

Figure 8. Functions of two variables used in multivariate equitability plot. The functions of two variables used to generate the multivariate equitability plots, before Gaussian noise is added. R code defining these functions is available from the authors upon request.

6 Significance tests.

To calculate a p-value to assess whether a set of observations departs significantly from independence, we use the cross-validation likelihoods for both the null and alternative models to produce a cross-validation likelihood ratio statistic (cvLRS):

$$cvLRS = -2\log\left[\frac{L_{cv}(null)}{L_{cv}(alt)}\right] \tag{6}$$

As with a traditional likelihood ratio test, we can compare the value of the cvLRS for the set of observations of interest against the distribution of the cvLRS that we expect if the null hypothesis is true and the variables are independent. We obtain the null distribution by drawing random permutations of the original data, where the permutations enforce independence. The significance test associated with $\hat{A}$ was run on a number of different distributions (see figure 9). As comparators, we used the dCov test [2], the test associated with MIC [3], and a state-of-the-art test by Heller, Heller and Gorfine (HHG) for all departures from independence [4]. The results paint a complex picture (see figures 10 and 11). There is no clear victor, and the relative performance depends strongly on the form of the distribution. Overall, the $\hat{A}$ test sometimes greatly outperforms all other tests, but it is never far from the best test (except for the purely linear Gaussian case, in which dCov has an advantage). This is in contrast with MIC, which has very low power across all sample sizes for 4 of the 7 non-independent distributions. This lack of power has already been pointed out in commentary by Simon and Tibshirani (`www-stat.stanford.edu/~tibs/reshef/comment.pdf`) and by Gorfine, Heller and Heller (`iew3.technion.ac.il/~gorfinm/files/science6.pdf`).

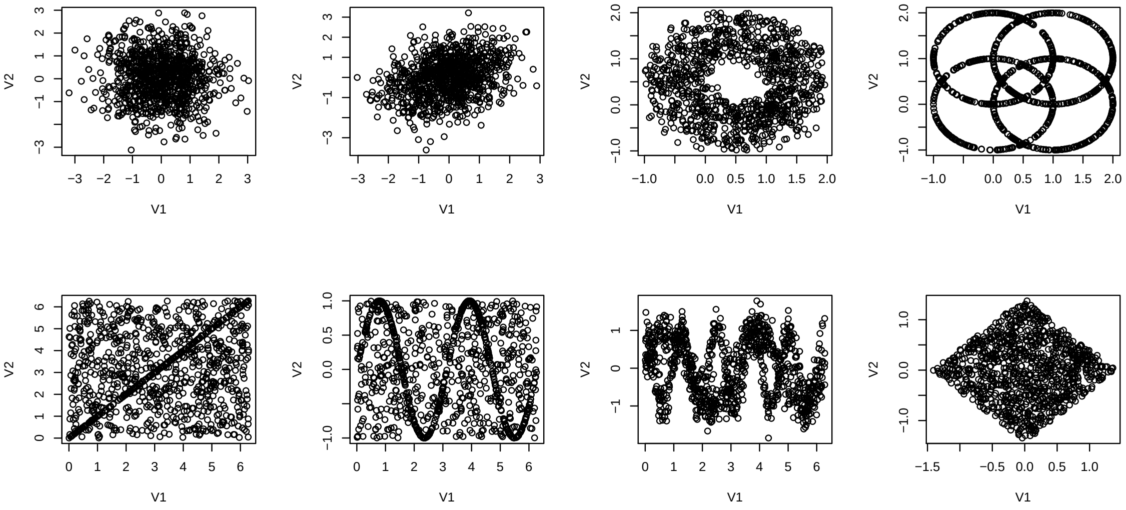

Figure 9. The distributions used to compare the power of some tests of significance. Each of these plots was created using $n = 1000$ to visualize the distributions, although the tests were conducted with smaller sample sizes. We adjusted the amount of noise to give the power of the tests a meaningful dynamic range for the sample sizes we considered. Very complex relationships like the four overlapping circles required no noise at all, whereas a single circle required much more noise to provide a challenging example. Starting clockwise from top left, the distributions are: 1) independent Gaussian noise, 2) slightly correlated Gaussian noise ($R^2 = 0.1$), 3) a circle with Gaussian noise added to both dimensions 4) 4 noiseless overlapping circles, 5) a uniform diamond, 6) a mixture of noisy sinusoids, 7) a noiseless sinusoid against a background of uniform noise and 8) a noiseless straight line against a background of uniform noise.

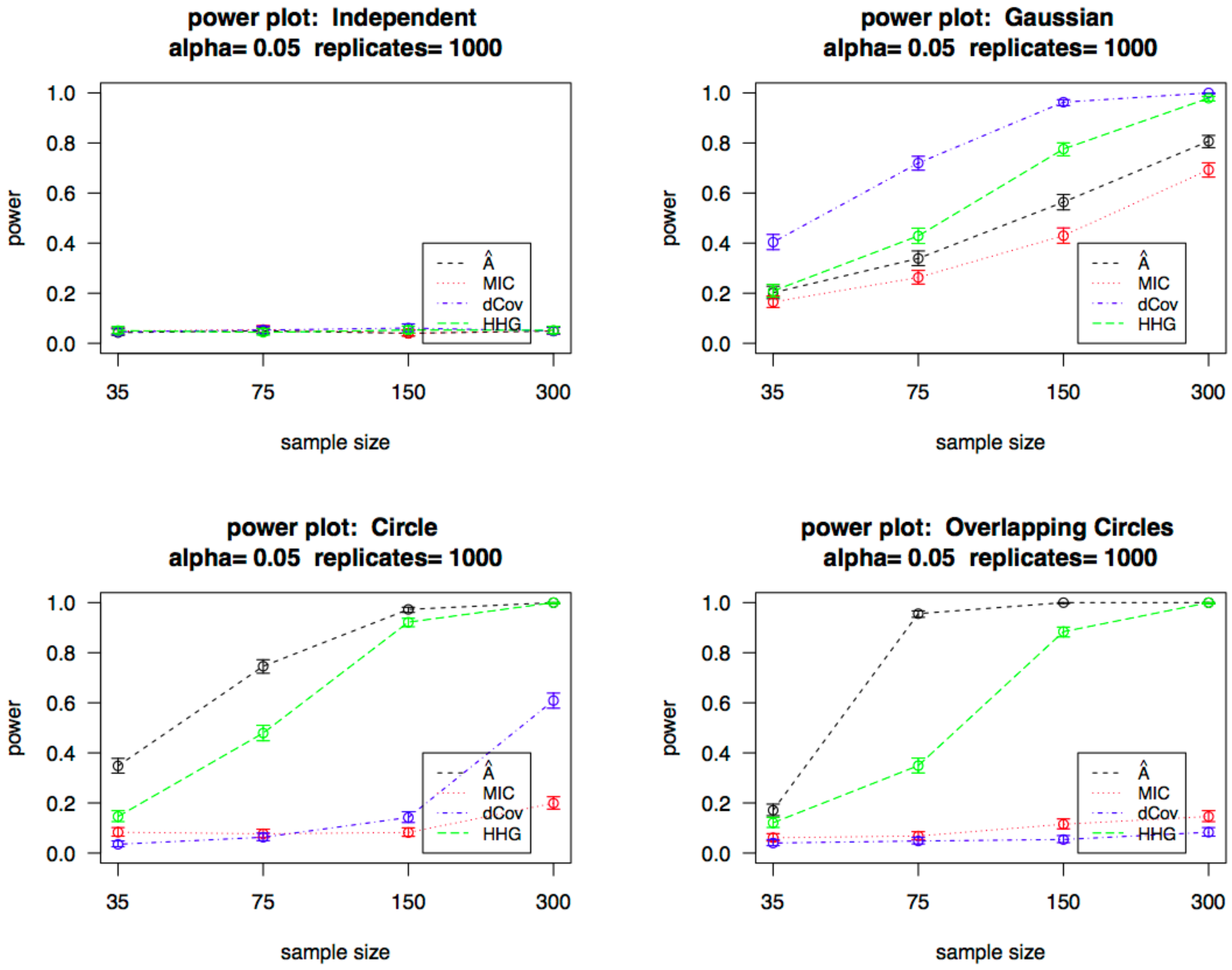

Figure 10. Power curves for increasing sample sizes for the distributions from the top row of figure 9. On independent data, all tests produce false positives in line with the selected test size, $\alpha = 0.05$. For a weak linear association, the dCov test outperforms the others. For the noisy circle and the 4 overlapping circles, the $\hat{A}$ test dominates. Error bars are 95% binomial confidence intervals (Wilson's method).

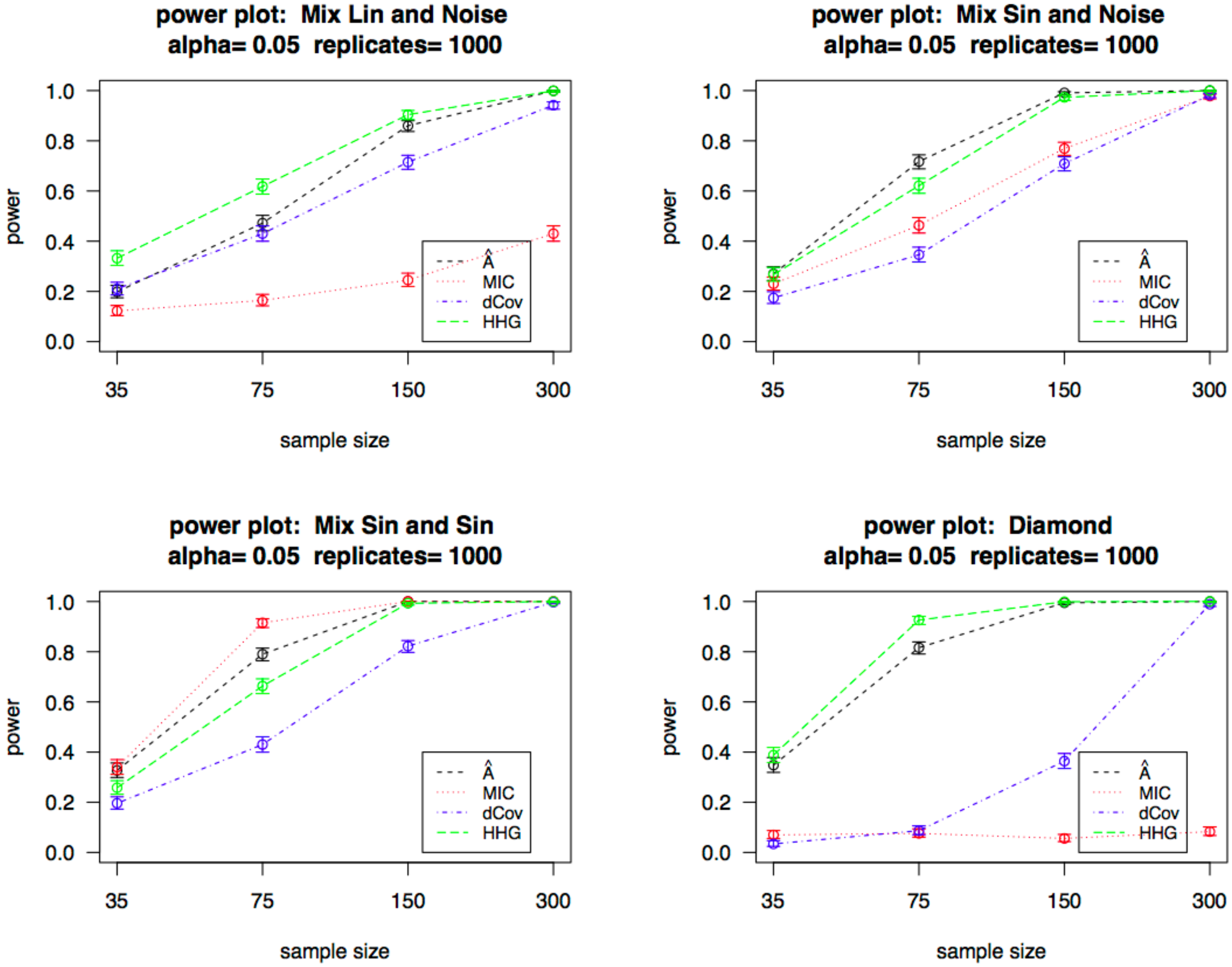

Figure 11. Power curves for increasing sample sizes for the distributions from the bottom row of figure 9. HHG has the greatest power when a linear relationship is obscured by independent background noise. The $\hat{A}$ test is superior when a sinusoid is placed against a noisy background. MIC outperforms the $\hat{A}$ test for a mixture of noisy sinusoids when $N = 75$, and is equal for other samples sizes. HHG slightly outperforms the $\hat{A}$ test on the diamond, where dCov's power grows slowly with sample size and MIC has uniformly low power. Error bars are 95% binomial confidence intervals (Wilson's method).

7 $\hat{A}$ can detect manifolds.

If all data points lie along a lower dimensional non-trivial manifold in a higher dimensional space (by non-trivial we mean that the manifold cannot be elucidated by merely discarding a variable), then $\hat{A}$ appears to tend to 1 as the sample size increases. We demonstrate this manifold detection property of $\hat{A}$ using data scattered on simple manifolds embedded in 3 dimensions.

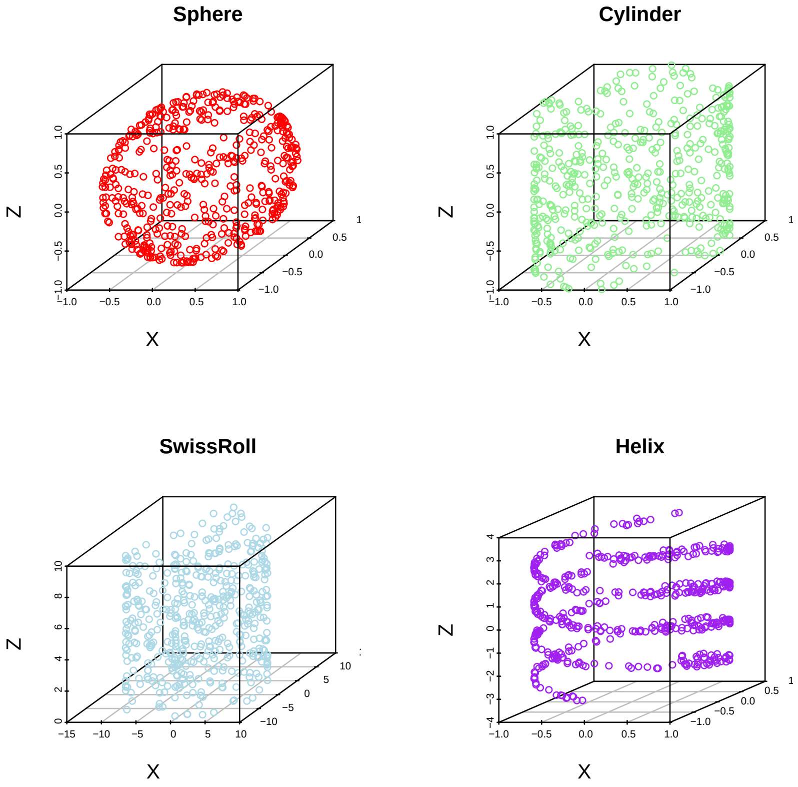

Figure 12. Manifolds detection examples. Examples of the point clouds used in the manifold detection exercise.

In figure 13 we compute $\hat{A}$ on the $3D$ datasets of figure 12 using a 3 part partition, computing the alternative likelihood as $\approx P(V_1, V_2, V_3)$, but the null as $\approx P(V_1)P(V_2)P(V_3)$. $\hat{A}$ approaches unity, indicating that there is some noiseless relationship between these sets of variables.

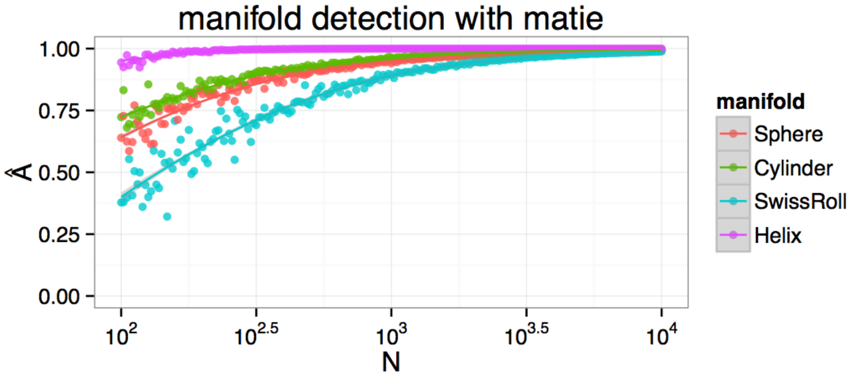

Figure 13. **$\hat{A}$ approaches unity as sample size increases in the presence of a manifold.** The helix is detected at the lowest sample sizes, perhaps because it is a 1 dimensional manifold in 3 dimensions, whereas the other examples are 2 dimensional manifolds in 3 dimensions.

8 Semipartial association - controlling for variables.

When the data are truly linear, $\hat{A}_{Y,X;C}$ yields very similar estimates to the linear $R^2_{Y,X;C}$ (estimated using the R function 'spcor'). We generated data where $X \sim \mathcal{N}(0,1)$, $C \sim \mathcal{N}(0,1)$, and $Y \sim k*X+(1-k)*C+\mathcal{N}(0,\epsilon)$, where k controls how much of the variance in Y is governed by X and how much by C, and ϵ is a small constant to prevent the linear package from encountering a singularity. Thus $P(Y,X,C)$ is jointly normally distributed. When we vary k from 0 to 1, $\hat{A}_{Y,X;C}$ yields strikingly similar estimates to $R^2_{Y,X;C}$, as seen in figure 14.

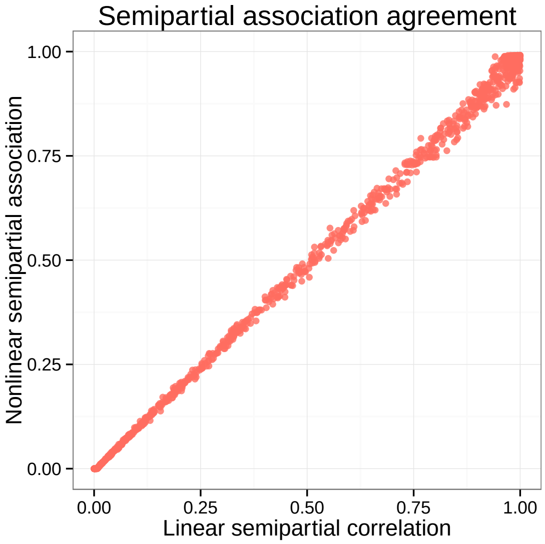

Figure 14. Agreement between linear semipartial correlation, and nonlinear semipartial association for linear data. The x axis depicts the linear $R^2_{Y,X;C}$ as estimated by the 'spcor' function in R, and the y axis depicts the nonlinear $\hat{A}_{Y,X;C}$. Each red point is the semipartial association estimated two ways from a simulated dataset, with 500 points drawn from a jointly Gaussian distribution, varying the strength of the semipartial correlation.

9 Example: BEAST analysis.

To demonstrate the abilities of $\hat{A}$ on non-contrived data, we analyse the relationships between variables comprising the posterior distribution of a BEAST Markov Chain Monte Carlo (MCMC) analysis. BEAST [5] is a Bayesian phylogenetics package that models the evolution of gene sequences over an unknown evolutionary history. A 'model' comprises a phylogeny and a number of model parameters, such as mutation rates, nucleotide frequencies, the ages of internal nodes in the phylogeny, and more. A likelihood function is constructed to explain the sequence data and, where available, the dates of the observed taxa or internal nodes. Using MCMC, BEAST draws samples from the posterior distribution of the model parameter conditioned on the observed data. These samples, along with whatever other auxiliary variables the user is interested in, are stored in the MCMC "chain". For our purposes, we will treat samples from the posterior as data to quantify the associations between variables. Here, we examine the BEAST analysis of a number of Influenza sequences from Wertheim *et al.* [6], thinning the MCMC chain to 1000 samples.

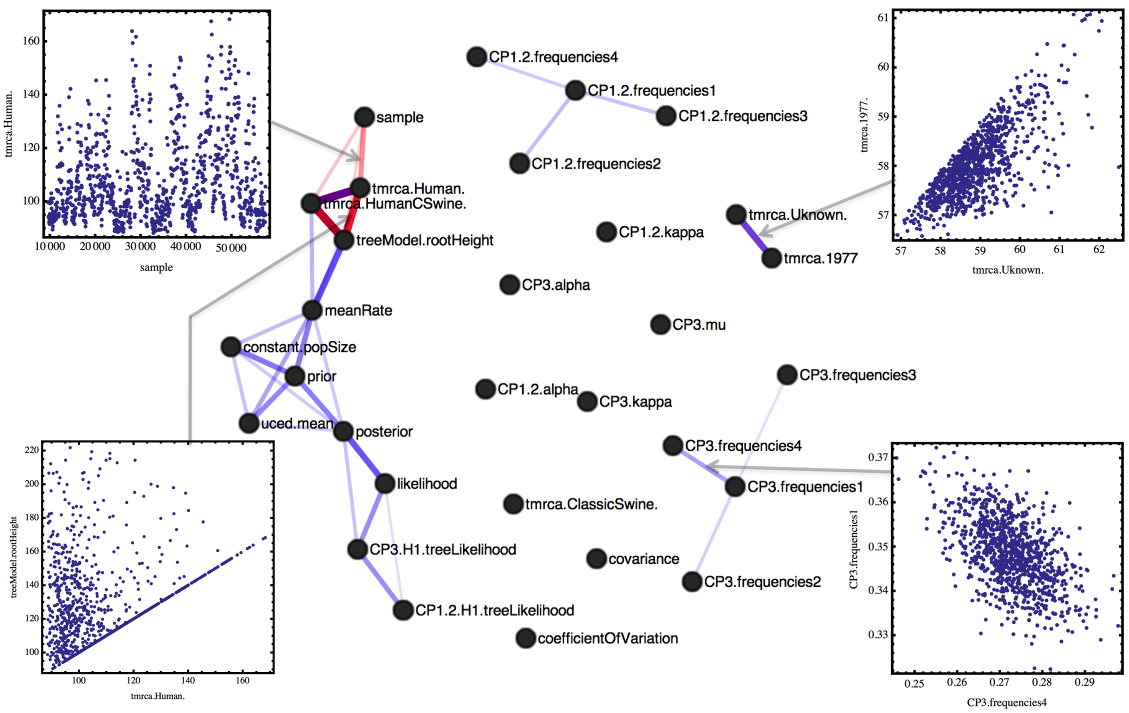

Figure 15. Network of pairwise associations form a BEAST analysis. $\hat{A}$ was used to quantify the association between variables from the posterior distribution of a BEAST analysis.

Fig. 15 depicts a pairwise examination of the associations between all variables, visualised using a force-directed graph (using the standard D3 javascript implementation: `http://d3js.org/`). Stronger associations are pulled together by shorter, wider, and bolder links (removing links where $\hat{A} < 0.1$). Links

are coloured by their degree of non-linearity from red to blue to depict non-linear to linear relationships, respectively. Some variables do not covary substantially with others, and are seen as isolated points. Of the relationships identified, most are linear, and they tend to occur in clusters of related variables, but there are a few non-linear relationships. We expand the scatter plots of four example relationships. First, the pairwise associations between 'tmrca.Human' (the Time to Most Recent Common Ancestor of the Human clade), and 'treeModel.rootHeight' is strongly non-linear ($\hat{A} = 0.81$, linear $\rho^2 = 0.06$). Examining the scatter plot indicates that there appears to be a superposition of a perfect linear relationship with a weaker linear relationship. This can be explained by BEAST marginalising over uncertainty in the phylogeny itself, so that, for some parts of the MCMC chain, the MRCA for the human clade *was* the root of the tree, so their heights are identical, but for other parts of the tree some other node was occupying the root location. The next interesting non-linear relationship occurs between 'sample' and 'tmrca.Human' ($\hat{A} = 0.37$, linear $\rho^2 = 0.03$); 'sample' denotes the index of the MCMC chain, so if some variable is particularly strongly related to 'sample', then it indicates that the chain is mixing particularly slowly with respect to that variable. This would usually be quantified using a measure of autocorrelation, but we note that with $\hat{A}$ the non-linear dependence is identified in the raw relationship, without having to explicitly compare values to their successors, as is done to analyse autocorrelation. We also depict the mildly non-linear 'tmrca.Unknown' vs 'tmrca.1977' ($\hat{A} = 0.63$, $\rho^2 = 0.55$), and the completely linear 'CP3.frequencies4' vs 'CP3.frequencies1' ($\hat{A} = 0.29$, $\rho^2 = 0.29$).

$\hat{A}$ can identify higher order relationships, such as the triplet association score, using the likelihood of a full joint $P(V_1, V_2, V_3)$ against a null of $P(V_1)P(V_2)P(V_3)$. We computed all triplet relationships, and ranked them by the difference between the triplet $\hat{A}$ score and the greatest pairwise $\hat{A}$ score (see table 1 below for the first 20). Two near-perfect triplet relationships are identified with $\hat{A} = 0.99$. Both are linear, and straightforward to explain. The relationship between 'posterior', 'prior', and 'likelihood' is simply a result of Bayes' theorem, while the relationship between 'likelihood', 'CP1.2.H1.treeLikelihood' and 'CP3.H1.treeLikelihood' simply reflects the fact that the overall log-likelihood is the sum of the log-likelihood from the first and second codon positions (which share parameters) and from the third codon position (which uses different parameters). Further down the list (at positions 5, 6, 8 and 9) are relationships between triplets of nucleotide frequency variables, with triplet $\hat{A}$ scores ranging from 0.36 to 0.51. This reflects the fact that the sum over nucleotide frequencies should be 1, inducing a relationship that A identifies. In fact, when we include all 4 nucleotide frequencies, the association gets much stronger: 0.91 for the merged first two codon positions and 0.93 for the third codon position. These association scores approach 1 as the sample size increases (they are both 0.98 for a sample size of 11305, obtained by less stringent thinning of the MCMC chain), which reflects a tendency of $\hat{A}$ to act conservatively, especially in higher dimensions.

Table 1. BEAST triplet score table.

triplet $\hat{A}$	best pair $\hat{A}$	difference[a]	name V1	name V2	name V3
0.9892365	0.3563312	0.6329053	likelihood	CP1.2.H1.treeLikelihood	CP3.H1.treeLikelihood
0.9908218	0.5390134	0.4518084	posterior	prior	likelihood
0.6115287	0.3563312	0.2551975	posterior	CP1.2.H1.treeLikelihood	CP3.H1.treeLikelihood
0.5069893	0.2879703	0.2190190	CP3.frequencies1	CP3.frequencies2	CP3.frequencies4
0.4251341	0.2064465	0.2186875	CP1.2.frequencies1	CP1.2.frequencies2	CP1.2.frequencies4
0.7651117	0.5702889	0.1948228	prior	treeModel.rootHeight	meanRate
0.3759378	0.2064465	0.1694912	CP1.2.frequencies1	CP1.2.frequencies2	CP1.2.frequencies3
0.4507804	0.2879703	0.1628100	CP3.frequencies1	CP3.frequencies3	CP3.frequencies4
0.5842424	0.4308488	0.1533936	posterior	prior	constant.popSize
0.5803565	0.4282539	0.1521025	prior	uced.mean	meanRate
0.5828469	0.4308488	0.1519980	prior	constant.popSize	meanRate
0.5406853	0.3955828	0.1451025	posterior	prior	uced.mean
0.5697743	0.4282539	0.1415204	posterior	prior	meanRate
0.6762243	0.5390134	0.1372109	posterior	likelihood	constant.popSize
0.3275699	0.1920072	0.1355627	CP1.2.frequencies1	CP1.2.frequencies3	CP1.2.frequencies4
0.5295651	0.3955828	0.1339822	posterior	prior	CP3.H1.treeLikelihood
0.5645590	0.4308488	0.1337101	prior	constant.popSize	uced.mean
0.6524119	0.5390134	0.1133985	posterior	likelihood	meanRate
0.6760031	0.5702889	0.1057142	treeModel.rootHeight	uced.mean	meanRate
0.6425790	0.5390134	0.1035656	posterior	likelihood	uced.mean

[a]The difference between the triplet $\hat{A}$ score and the highest pairwise $\hat{A}$. The table is sorted by this value.

10 MIC can return 1 for noisy relationships.

MIC is supported by a theorem that guarantees a value of 1 when a relation is noiseless and nowhere flat. Such a guarantee less useful if MIC can achieve 1 for noisy relationships as well, as demonstrated in figure 16.

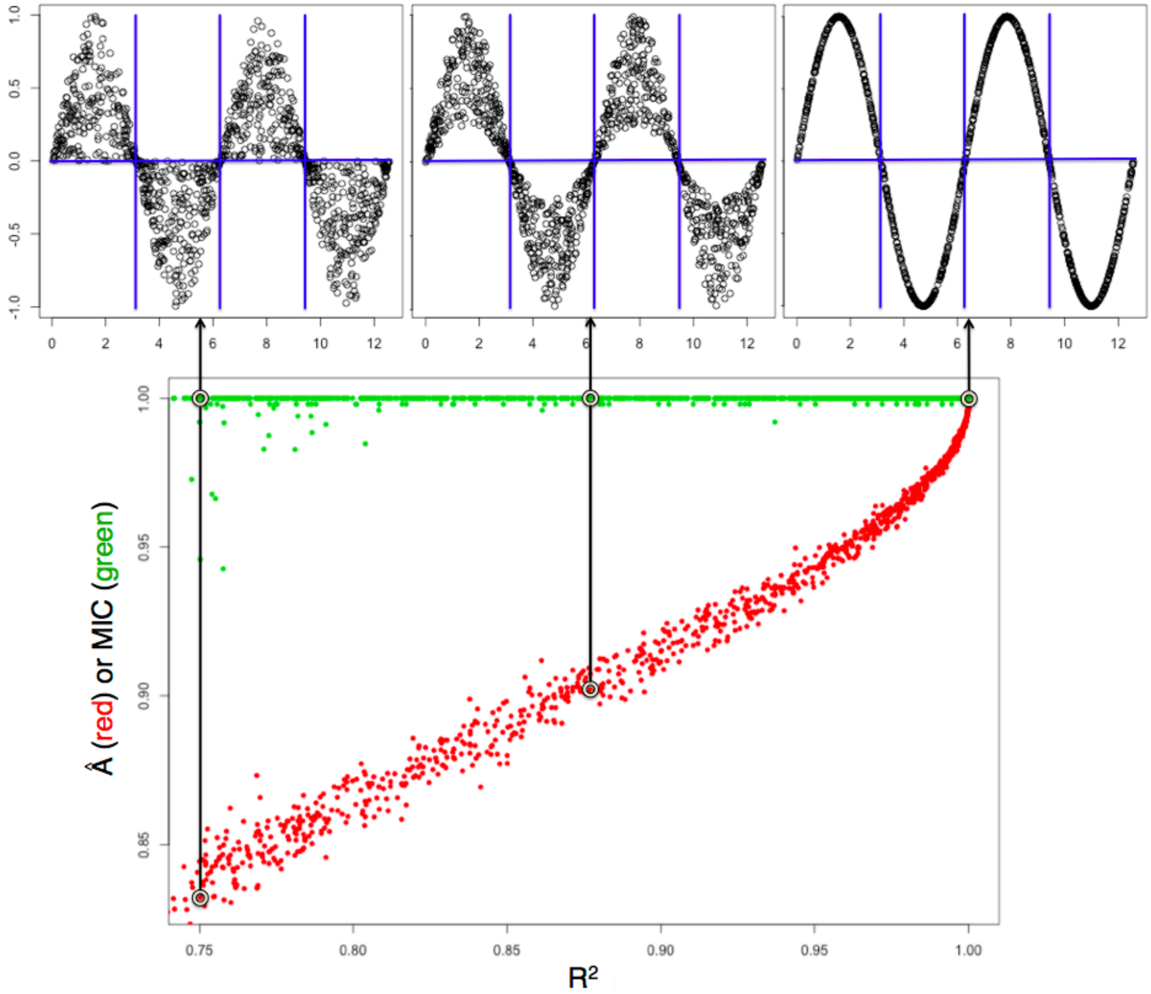

Figure 16. A pathology caused by MIC's grid. When data are generated by a sinusoid with uniform multiplicative noise ($X \sim U(0, 4\pi)$, $Y \sim \sin(X) \times U(k, 1)$, where k lies between 0 and 1), MIC assigns scores of 1 (green) irrespective of the level of noise (we suspect that few values less than one are due to the approximations employed when searching for the optimum grid). This is because MIC can find an optimal grid (blue) that is overly coarse, and the structure of the data within the grid cells is ignored. $\hat{A}$, on the other hand, performs appropriately on this example. Depicted above are examples at various values of R^2.

11 $\hat{A}$ is robust to outliers.

See figure 18 for plots. Data points (n=100) were first generated from a bivariate normal distribution, with the standard deviations of X and $Y = 1$. R^2 of the generating distribution (shown on the X axis of all plots) was varied incrementally from 0 to 1. Each association measure (Pearson, Spearman, MIC and $\hat{A}$) was calculated on each dataset. This measures the association without outliers (marked "Clean" on the plots). Then outliers are introduced by replacing a small number of points (3 for "Few" and 10 for "Many") with points drawn from a bivariate uniform distribution, whose range is (-2,+2) for "Close" and (-5,+5) for "Far". "Close" outliers tend to have a similar range to the distribution of interest (the bivariate normal), but "Far" outliers may fall outside of it. We overlay the outlier-contaminated scatter plots with the "Clean" ones to see the magnitude of the effect of outliers on the association measures. $\hat{A}$ and MIC are relatively robust to outliers, Spearman's correlation is moderately robust, and Pearson's is not robust at all. Loess curves are included as a guide for the eye.

Figure 17 shows the distribution of the differences in association between clean and contaminated data for a particular combination of generating parameters, overlaying the distributions for different methods to facilitate direct comparison.

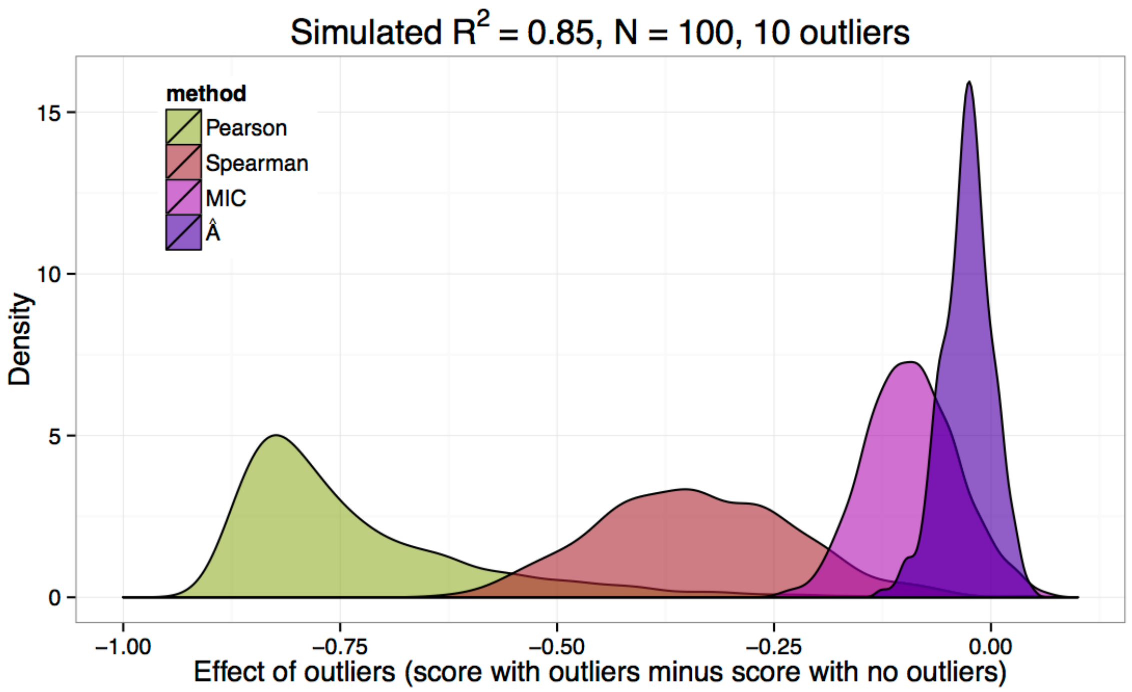

Figure 17. The distribution of differences between clean and contaminated data. Using a fixed generating $R^2 = 0.85$ and an outlier mechanism corresponding to "Many" and "Far" in figure 18 (the most extreme case), we display the difference between the association measured from the clean data and that from the outlier-contaminated data. $\hat{A}$ appears to have the smallest differences, followed by MIC, then Spearman's correlation, then Pearson's.

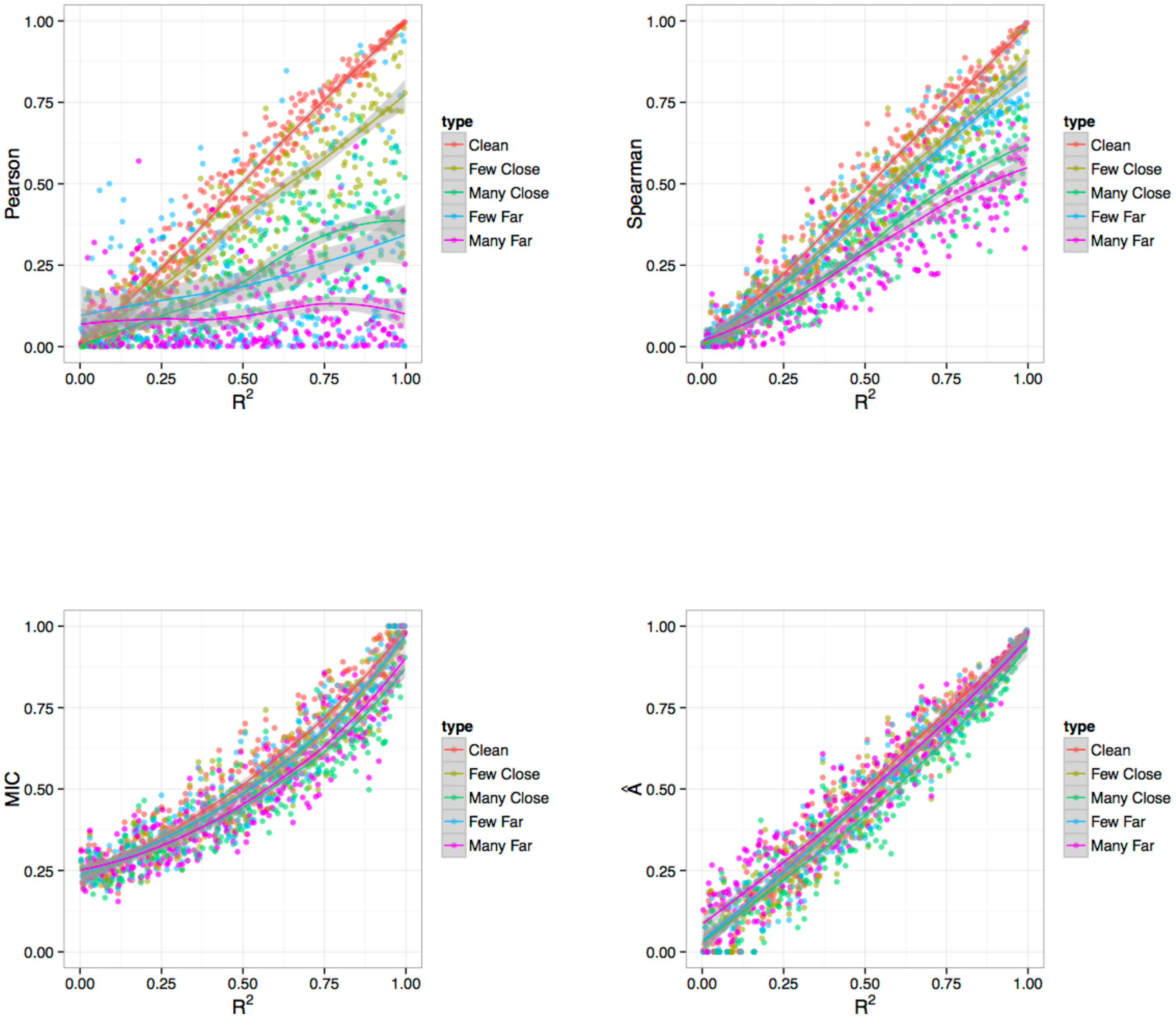

Figure 18. Robustness to outliers. “Clean”: associations measured with no outliers. “Close”: outliers have range similar to data. “Far”: outlier range extends beyond data range. “Few”: 3 outliers included. “Many”: 10 outliers included. See SI8 text for details.

12 A small samples bias correction.

Empirically, the raw estimates of $\hat{A}$ (when estimating the density) tend to converge to A (when the density is known) from below as the sample size increases, underestimating the strength of relationships for small samples. Providing conservative estimates when a lack of data precludes confidence in a strong relationship could be interpreted as an attractive feature. This might not be ideal for all applications, so we sought to correct this bias. We postulated (aided by inspection) the form of a correction - the amount by which to adjust the alternative model likelihood - and simulated bivariate Gaussian data from a range of sample sizes and association strengths, finding the parameter values for the correction that maximized the agreement between the true association (ρ^2 of the bivariate Gaussian) and $\hat{A}$.

The alternative log likelihoods were offset using the following correction:

$$\left(1 - \frac{1}{1 + T\hat{A}}\right) \frac{N^P}{S}$$

and $\hat{A}$ was then re-estimated using the adjusted alternative log likelihood.

The three parameters of the correction, T, S and P, were optimized to minimize the mean squared deviation from the known R^2. See figure 19 for an example comparing the corrected and uncorrected values. All results in main text and the SI use this correction - the default in the matie package.

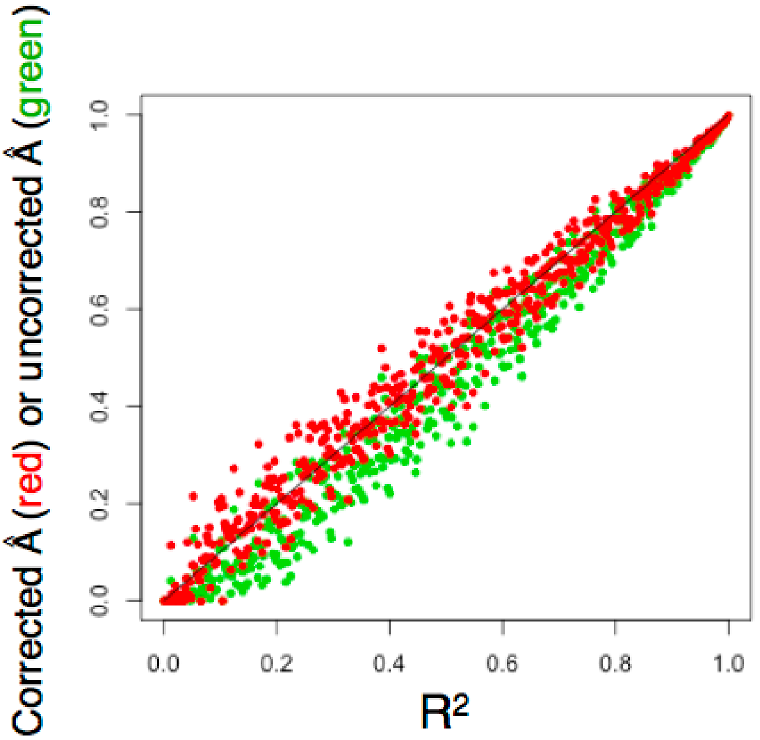

Figure 19. Bias correction. Data were generated from a bivariate Gaussian distribution ($N = 200$) with R^2 ranging from 0 to 1, and the uncorrected (green) and corrected (red) $\hat{A}$ estimates are displayed.

13 `matie`, An R package for computing $\hat{A}$.

$\hat{A}$ can be estimated using an R package, `matie` (Measuring Associations and Testing Independence Efficiently), available on CRAN (`http://cran.r-project.org/web/packages/matie/`).

The main function of `matie` is `ma` (measure association). This function computes associations between between any number of variables, each of which may be vector valued. The canonical example discussed in the main text is the bivariate case, X against Y. Another example from the main text is the "one against two" case, where the association is computed between a scalar valued X and a vector valued $\mathbf{Y}$, with components $\langle Y_1, Y_2 \rangle$. While the description in Methods (from the main text) only handles the case for two (possibly vector valued) variables, it naturally extends beyond that to any number of vector valued variables. We use this in SI7 to detect lower dimensional manifolds embedded in a higher dimensional space. The principle is the same: the likelihood under a full joint density model is compared to that of the product of marginal densities.

The function `ma` takes a dataset, in the form of a matrix, where each row is an observation. Each observation is a list of real values, but `ma` needs to know which values belong to which vector valued variables. In the package, this is implemented in the form of a 'partition', which assigns values to variables. For example, if each observation has 3 values, a partition of $(\langle 1 \rangle, \langle 2, 3 \rangle)$ will compute the proportion of variance in the first variable that can be explained by the combination of the second and third variables (as in figure 4 from the main text). In this case, the alternative model will be the joint of all three $\approx P(V_1, V_2, V_3)$, but the null will be the product of the marginal of the first with the joint of the rest, $\approx P(V_1)P(V_2, V_3)$. For a partition of $(\langle 1 \rangle, \langle 2 \rangle, \langle 3 \rangle)$ (as in SI7), matie will compute the alternative as $\approx P(V_1, V_2, V_3)$, but the null as $\approx P(V_1)P(V_2)P(V_3)$. Another way of thinking about this is that the partition specifies the factorization of the null model.

The `matie` package also provides functionality that allows the user to:

- compute p-values testing against the null hypothesis of independence
- compute the non-linearity in a relationship
- compute the semipartial association between two variables, controlling for a third covariate
- visualize associations and non-linearity in datasets
- simulate data to investigate the behavior of the methods

14 Execution time: matie versus MIC.

MIC (using the Java package provided by the authors) and $\hat{A}$ (using matie) were computed for a number of different sample sizes: n= 50, 100, 200, 400, 800, 1600, 3200. Each point in figure 20 is the computation time taken to compute a single bivariate association, averaged over 45 replicates. The curves are 0 intercept quadratics (fit with least squares), and the empirical computation times appear to be quadratic for both methods. All computation times were measured on a 2012 Apple MacBook Air.

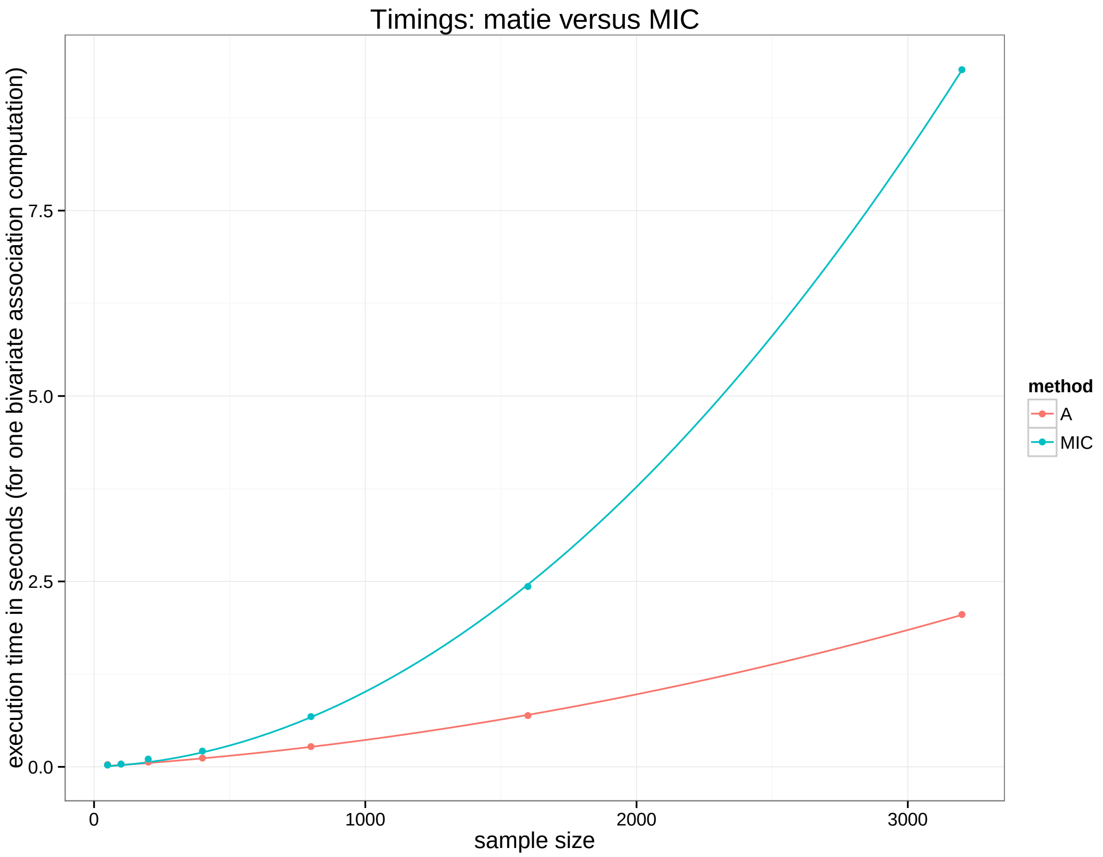

Figure 20. Comparison between $\hat{A}$ and MIC execution times. The curves are 0 intercept quadratics (fit with least squares), and the empirical computation times appear to be quadratic for both methods.

References

1. Linfoot EH. An informational measure of correlation. Information and Control. 1957;1(1):85–89.

2. Székely GJ, Rizzo ML. Brownian distance covariance. The Annals of Applied Statistics. 2009;3(4):1236–1265.

3. Reshef DN, Reshef YA, Finucane HK, Grossman SR, McVean G, Turnbaugh PJ, et al. Detecting Novel Associations in Large Data Sets. Science. 2011;334(6062):1518–1524.

4. Heller R, Heller Y, Gorfine M. A consistent multivariate test of association based on ranks of distances. Biometrika. 2012;.

5. Drummond AJ, Suchard MA, Xie D, Rambaut A. Bayesian phylogenetics with BEAUti and the BEAST 1.7. Molecular biology and evolution. 2012;29(8):1969–1973.

6. Wertheim JO. The Re-Emergence of H1N1 Influenza Virus in 1977: A Cautionary Tale for Estimating Divergence Times Using Biologically Unrealistic Sampling Dates. PLoS ONE. 2010;5(6):e11184+.
